# Finding emergence in data by maximizing effective information

**DOI:** 10.1093/nsr/nwae279

**Published:** 2024-08-12

**Authors:** Mingzhe Yang, Zhipeng Wang, Kaiwei Liu, Yingqi Rong, Bing Yuan, Jiang Zhang

**Affiliations:** School of Systems Science, Beijing Normal University, Beijing 100875, China; School of Systems Science, Beijing Normal University, Beijing 100875, China; School of Systems Science, Beijing Normal University, Beijing 100875, China; Department of Cognitive Science, Johns Hopkins University, Baltimore 21218, USA; Swarma Research, Beijing 102300, China; School of Systems Science, Beijing Normal University, Beijing 100875, China; Swarma Research, Beijing 102300, China

**Keywords:** causal emergence, dynamics learning, effective information, coarse graining, invertible neural network

## Abstract

Quantifying emergence and modeling emergent dynamics in a data-driven manner for complex dynamical systems is challenging due to the fact that emergent behaviors cannot be directly captured by micro-level observational data. Thus, it is crucial to develop a framework to identify emergent phenomena and capture emergent dynamics at the macro-level using available data. Inspired by the theory of causal emergence (CE), this paper introduces a machine learning framework to learn macro-dynamics in an emergent latent space and quantify the degree of CE. The framework maximizes effective information, resulting in a macro-dynamics model with enhanced causal effects. Experimental results on simulated and real data demonstrate the effectiveness of the proposed framework. It quantifies degrees of CE effectively under various conditions and reveals distinct influences of different noise types. It can learn a one-dimensional coarse-grained macro-state from functional magnetic resonance imaging data to represent complex neural activities during movie clip viewing. Furthermore, improved generalization to different test environments is observed across all simulation data.

## Teaser

This work presents a machine learning framework for quantifying causal emergence and modeling macro-dynamics in complex systems, effectively capturing emergent phenomena from data and enhancing generalization across diverse conditions.

## INTRODUCTION

The climate system, ecosystems, bird flocks, ant colonies, cells, brains and many other complex systems are composed of numerous elements and exhibit a wide range of complex behaviors [1,2]. In the past few decades, the research topic of data-driven modeling in complex systems has gained significant attention, driven by the increasing availability and accumulation of data from real dynamical systems [3–5]. However, complex systems always exhibit emergent behaviors [1]. That means some interesting emergent patterns or dynamical behaviors such as waves [6], periodic oscillations [7] and solitons [8] can hardly be directly observed and identified from the micro-level behavioral data. Therefore, the identification and measure of emergence and the capture of emergent dynamical patterns solely from observational raw data have become crucial challenges in complex system research [9,10]. But, in order to address these problems, it is necessary to first develop a quantitative understanding of emergence.

**Figure 1.**
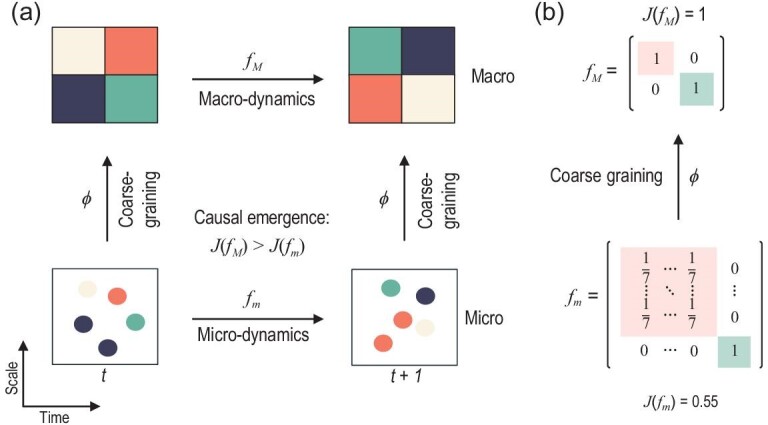
(a) An illustration of the fundamental concept of causal emergence (CE) in [19]. The effective information (EI) is denoted as $\mathcal {J}$ in this paper. (b) A case demonstrating CE in a discrete Markov chain. The micro-dynamics consists of eight micro-states. During the coarse-graining process, the first seven states are grouped together as one macro-state, while the eighth micro-state corresponds to the second macro-state. As a result, a transition probability matrix is formed at the macro-scale, where the effective information $\mathcal {J}(f_M)=1$ (calculated using Equation S1), which is greater than $\mathcal {J}(f_m)=0.55$. This difference, $\Delta \mathcal {J}=0.45$, indicates the occurrence of CE, as $\Delta \mathcal {J}> 0$.

Emergence, as a distinctive feature of complex systems [11], has historically been challenging to quantify and describe in quantitative terms [12,13]. Most conventional measures or methods either rely on pre-defined macro-variables (e.g. [14–16]) or are tailored to specific scenarios in engineered systems (e.g. [17,18]). However, there is a need for a unified method to quantify emergence across different contexts. The theory of causal emergence (CE) [19,20] offers a framework to tackle this challenge. Hoel *et al.* [19] aimed to understand emergence through the lens of causality. The connection between emergence and causality is implied in the descriptive definition of emergence, as stated in the work of Fromm [21]. According to this definition, a macro-level property, such as patterns or dynamical behaviors, is considered emergent if it cannot be explained or directly attributed to the individuals in the system. The theory of causal emergence formalizes this concept within the framework of discrete Markov dynamical systems. As shown in Fig. 1a, Hoel *et al.* [19] stated that if a system exhibits stronger causal effects after a specific coarse-graining transformation compared to the original system then CE has occurred. In [19], the degree of causal effect in a dynamical system is quantified using effective information (EI) [22]. EI can be understood as an intervention-based version of mutual information between two successive states in a dynamical system over time. It is a measure that solely depends on the system’s dynamics. If a dynamical system is more deterministic and non-degenerate, meaning that the temporal adjacent states can be inferred from each other in both directions of the time arrow, then it will have a larger EI [19]. This measure has been shown to be compatible with other well-known measures of causal effects [23]. Figure 1b gives an example of CE for the simple Markov chain.

While causal emergence theory has successfully quantified emergence using EI and has found applications in various fields [24–26], there are some drawbacks. Firstly, the Markov transition matrix of the micro-dynamics should be given rather than constructed from data. Secondly, a pre-defined coarse-graining strategy must be provided or optimized through maximizing EI, but this optimization process is computationally complex [24,27]. Although Rosas *et al.* [9] proposed a new framework for CE based on partial information decomposition theory [28], which does not require a pre-defined coarse-graining strategy, it still involves iterating through all variable combinations on the information lattice to compare synergistic information, resulting in significant computational complexity. Rosas *et al.* [9] also proposed an approximate method to mitigate the complexity, but it requires a pre-defined macro-variable. Further details regarding the framework are available in Section S1.2. In addition, Barnett and Seth [29] introduced a novel framework for quantifying emergence based on the concept of dynamical independence. If the micro-dynamics are unrelated to the prediction of macro-dynamics, the complex system is considered to exhibit emergent macroscopic processes. They utilized transfer entropy to measure the correlation between macro-dynamics and micro-dynamics, thereby eliminating the need to obtain the Markov transition matrix. It also offers a method for identifying macro-variables. However, this framework has only been applied to linear systems to date, and it remains a challenge to extend the methods to more complex scenarios.

Therefore, the challenge of finding emergence in data, which involves determining whether CE has occurred within a system and to what extent based solely on observational data of its behavior, remains unresolved. The most daunting task is that all the elements, including the Markov dynamics at both the micro- and macro-levels, as well as the coarse-graining strategy to obtain macro-variables, need to be learned from raw data and cannot be pre-defined in advance [10]. Once the learning process is completed, we can compare the strength of causal effects (measured by EI) in dynamics at different scales to identify CE from the data. Therefore, the problem of finding CE within [19] is essentially equivalent to the challenge of data-driven modeling within a coarse-grained space for complex systems [10]. Building models for complex systems at multiple coarse-grained levels within learned emergent spaces is of utmost importance for both identifying CE and conducting data-driven modeling in complex systems.

Recently, several machine learning frameworks have emerged for learning and simulating the dynamics of complex systems within coarse-grained latent or hidden spaces [30–34]. While these learning systems can capture emergent dynamics, they may not directly address the fundamental nature of CE, which entails stronger causality. According to Judea Pearl’s hierarchy of causality, prediction-based learning is situated at the level of association and cannot address the challenges related to intervention and counterfactuals [35]. Empirically, dynamics learned solely based on predictions may be influenced by the distributions of the input data, which can be limited by data diversity and the problem of over fitting models [36]. However, what we truly desire is an invariant causal mechanism or dynamics that are independent of the input data. This allows the learned mechanism or dynamics to be adaptable to broader domains, generalizable to areas beyond the distribution of training data and capable of accommodating diverse interventions [37–39]. Unfortunately, not many studies have explored the integration of causality and latent space dynamics to address the challenges of data-driven modeling in complex systems [40].

**Figure 2.**
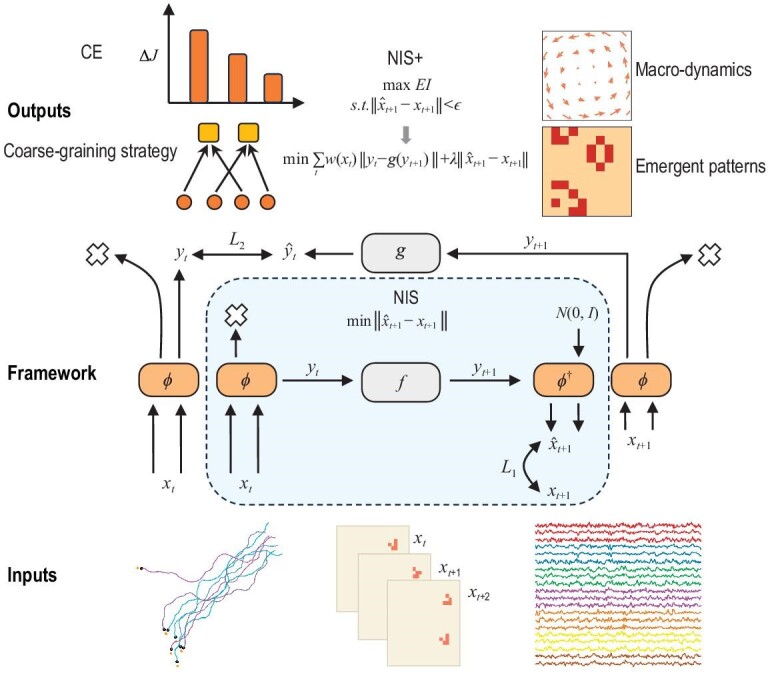
The architecture of our proposed framework, the neural information squeezer plus (NIS+), building upon our previous model, NIS [10,44]. It accepts a variety of time series as inputs and enables us to derive the degree of CE, the learned macro-dynamics, captured emergent patterns and the strategy for coarse graining. Within the framework, the boxes symbolize functions or neural networks, while the arrow pointing to a cross signifies the operation of information discarding. For further details regarding these mathematical symbols, see Section S2.2.

Inspired by the theory of causal emergence, this paper aims to address the challenge of learning causal mechanisms within a learned coarse-grained macro-level (latent) space. The approach involves maximizing the EI of the emergent macro-level dynamics, which is equivalent to maximizing the degree of causal effect in the learned coarse-grained dynamics [23]. To achieve this, a novel machine learning framework called the neural information squeezer plus (NIS+) is proposed. NIS+ extends the previous framework (NIS) to solve the problem of maximizing EI under coarse-grained representations. As shown in Fig. 2, NIS+ not only learns emergent macro-dynamics and coarse-grained strategies, but also quantifies the degree of CE from time series data and captures emergent patterns. Mathematical theorems ensure the flexibility of our framework in different application scenarios. Empirical tests underscore the proficiency of NIS+ in seizing emergent patterns and detecting CE across diverse scenarios, such as the SIR (susceptible $\rightarrow$ infective $\rightarrow$ removed or died) model [41], collective bird movement [42] and Conway’s Game of Life [43]. Moreover, NIS+ has been utilized to uncover emergent properties within actual neural data from 830 individuals engaged in a shared cinematic experience. These studies further confirm that the dynamical model derived from NIS+ shows superior generalization capabilities in comparison to alternative approaches.

## FINDING CAUSAL EMERGENCE IN DATA

Finding CE in time series data involves two sub-problems: *emergent dynamics learning* and *causal emergence quantification*.

### Problem definition

Suppose that the behavioral data of a complex dynamical system are a time series $\lbrace \boldsymbol{x}_t\rbrace$ with time steps $t=1,2,\dots ,T$ and dimension *p*, and that they form observable micro-states. The problem of *emergent dynamics learning* is to find three functions according to the data: a coarse-graining strategy $\phi :\mathcal {R}^p\rightarrow \mathcal {R}^q$, where $q\le p$ is the dimension of macro-states that is given as a hyperparameter; a corresponding anti-coarsening strategy $\phi ^{\dagger }:\mathcal {R}^p\rightarrow \mathcal {R}^q$ and a macro-level Markov dynamics $f_{q}$, such that the EI of macro-dynamics $f_q$ is maximized under the constraint that the predicted $\hat{\boldsymbol{x}}_{t+1}$ by $\phi$, $f_q$ and $\phi ^{\dagger }$ is closed to the real data of $\boldsymbol{x}_{t+1}$:


(1) \begin{eqnarray*}
&&\max _{\phi ,f_q,\phi ^+} \mathcal {J}(f_q)\\
&&\text{such that}
\left\{\begin{array}{c}
|| \hat{\boldsymbol{x}}_{t+1}-\boldsymbol{x}_{t+1} || < \epsilon ,\\
\hat{\boldsymbol{x}}_{t+1}=\phi ^{\dagger }(f_q(\phi (\boldsymbol{x}_t))).
\end{array}\right.
\end{eqnarray*}

Here $\epsilon$ is a given small constant and $\mathcal {J}$ is defined by


(2) \begin{eqnarray*}
\mathcal {J} = I(Y_t;\hat{Y}_{t+1}\mid {\rm do}(Y_t\sim U(\mathcal {Y}))),
\end{eqnarray*}

where $Y_t = \phi (X_t)$ and $\hat{Y}_{t+1} = f_q(Y_{t})$ represent the input and output variables of the macroscopic dynamics $f_q$, respectively. The notation ${\rm do}(Y_{t}\sim U(\mathcal {Y}))$ denotes the do operator [35], which intervenes on the system’s state at time *t* to force $Y_{t}$ to adhere to a uniform distribution across the value space $\mathcal {Y}$ of $Y_t$. For further details, see Sections S1.1 and S3.1.

The reason why we maximize EI in Equation (1) is to force the learned dynamics $f_q$ to have stronger causal effect. However, if we directly do this according to Fig. 1, a trivial solution will be obtained, as pointed by Zhang and Liu [10]. An exemplified trivial method involves mapping all micro-states to a value identical to that of the macro-state, maximizing macroscopic effective information ($\phi (x) = {\rm constant}$). Yet, this results in mere identical mapping, lacking causal emergence as all information is inherently lost. Therefore, we need to introduce constraints to avoid the problem. The constraints in Equation (1) imply that the macro-dynamics $f_q$ can simulate the micro-dynamics implied in the data as accurately as possible (the prediction error is less than a given threshold $\epsilon$).

By changing *q* we can obtain macro-dynamics in various dimensions. If $q=p$ then $f_p$ becomes the learnt micro-dynamics. Then we can compare $\mathcal {J}_q$ and $\mathcal {J}_p$ for any *q*. The problem of *causal emergence quantification* can be defined as the calculation of the difference


(3) \begin{eqnarray*}
\Delta \mathcal {J}\equiv \mathcal {J}(f_q)-\mathcal {J}(f_p),
\end{eqnarray*}

where $\Delta \mathcal {J}$ is defined as the degree of causal emergence. If $\Delta \mathcal {J}> 0$ then we say that CE occurs within the data.

### Solution

Solving the optimization problem defined in Equation (1) directly is difficult because all the optimized objects are functions and the objective function $\mathcal {J}$ is the mutual information after intervention that deserved a special process.

A novel machine learning framework called NIS+ has been developed to address this problem. The framework consists of two main components, with the details given in Fig. 2. The upper part focuses on optimizing the macro-dynamics *f* to minimize prediction errors at the micro-level, which satisfies the constraints described in Equation (1). This upper part is the original version of NIS. The lower part is specifically designed to optimize a reversed macro-dynamics *g*, which is crucial for optimizing mutual information, as outlined in Theorem 2.1 in Section S2.2.

The integration of these two parts forms NIS+, which simultaneously aims to optimize the forward macro-dynamics *f* and the reversed macro-dynamics *g*. Both parts share an encoder and a decoder, which model coarse-graining and anti-coarse-graining strategies, respectively. To reduce model complexity and the number of parameters, an invertible neural network is employed and the parameters are shared by $\phi$ and $\phi ^{\dagger }$ such that $\phi ^{\dagger }\approx \phi ^{-1}$.

Furthermore, to ensure that the optimized objective function is EI, we employ the *inverse probability reweighting technique* for $\mathbf {y}_t$. This technique allows us to simulate an intervention that forces $\mathbf {y}_t$ to follow a uniform (maximum entropy) distribution. The details are given in the section Methods and Data.

## RESULTS

We validate the effectiveness of the NIS+ framework through numerical experiments with data generated by different artificial models (dynamical systems, multi-agent systems and cellular automata). Additionally, we apply NIS+ to real functional magnetic resonance imaging (fMRI) data from human subjects to uncover interesting macro-level variables and dynamics. In these experiments, we evaluate the models’ prediction and generalization abilities. We also assess their capability to identify CE and compare it with the $\Psi$ indicator proposed in [9], an alternative measure for quantifying CE approximately.

### SIR

The first experiment revolves around a basic epidemiological dynamics model, the SIR model. In this experiment, the SIR dynamics serve as the ground truth for the macro-level dynamics, while the micro-level variables are generated by introducing noise to the macro-variables. The primary objective is to evaluate our model’s ability to effectively remove noise, uncover meaningful macroscopic dynamics, identify CE and demonstrate generalization beyond the distribution of the training dataset.

Formally, the macro-dynamics can be described as


**Figure 3.**
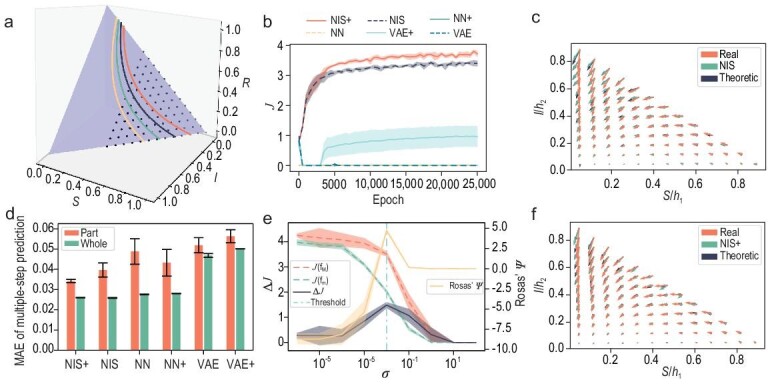
The experimental results of NIS+ and compared models on the SIR model with observational noise. (a) The phase space of the SIR model, along with four example trajectories with the same infection and recovery or death rates. The full dataset (entire triangular region) and the partial dataset (dotted area) used for training are also displayed. (b) The change in dimension-averaged effective information ($\mathcal {J}$) with training epochs in five repeated experiments. (c) A comparison is made among the vector fields of the SIR dynamics, the learned macro-dynamics of NIS+ and the macro-dynamics transformed by the Jacobian of the learned encoder. Each arrow represents a direction, and the magnitude of the derivative of the dynamics at that coordinate point. For detailed procedures, see Section S5.3. (d) A comparison is conducted to evaluate the errors in multi-step predictions for different models trained on either partial datasets or complete datasets. The heights and the error bars respectively denote the mean error and the standard deviation derived from five repetitions of the experiments conducted on the test set. For details on the parameters of these models, see Section S5.4; for data details, see Section S5.1. (e) The variations in the measure of CE ($\Delta \mathcal {J}$) and EI for micro-dynamics ($\mathcal {J}(f_m)$) and macro-dynamics ($\mathcal {J}(f_M)$) are plotted as the standard deviation $\sigma$ of observation noise changes. Following the definition in [9] and the calculation method for CE (see Section S3.1), the changes in $\Psi$ are calculated. The vertical line represents the threshold for the normalized MAE equaling 0.3. (f) A comparison is made among the vector fields of the SIR dynamics, the learned macro-dynamics of NIS and the macro-dynamics transformed by the encoder Jacobian matrix of NIS, in comparison with (c).

(4) \begin{eqnarray*}
\left\{\begin{array}{l}\displaystyle\frac{\mathrm{d}S}{\mathrm{d}t}=-\beta SI, \\
\displaystyle\frac{\mathrm{d}I}{\mathrm{d}t}=\beta SI - \gamma I, \\
\displaystyle\frac{\mathrm{d}R}{\mathrm{d}t}= \gamma I,\end{array}\right.
\end{eqnarray*}

where $S,I,R\in [0,1]$ represent the proportions of healthy, infected and recovered or died individuals in a population, and $\beta =1$ and $\gamma =0.5$ are parameters for infection and recovery rates, respectively. Figure 3a shows the phase space $(S,I,R)$ of the SIR dynamics. Because the model has only two degrees of freedom, as *S, I* and *R* satisfy $S+I+R=1$, all macro-states are distributed on a triangular plane in three dimensions, and only *S* and *I* are used to form the macro-state variable $\boldsymbol{y}=(S,I)$.

We then expand $\boldsymbol{y}$ into a four-dimensional vector and introduce Gaussian noises to form a microscopic state:


(5) \begin{eqnarray*}
\left\{\begin{array}{l}
\boldsymbol{S}^{\prime }=(S,S)+\boldsymbol{\xi }_1, \\
\boldsymbol{I}^{\prime }=(I,I)+\boldsymbol{\xi }_2.
\end{array}\right.
\end{eqnarray*}

Here $\boldsymbol{\xi }_1,\boldsymbol{\xi }_2 \sim {N}(0,\Sigma )$ are two-dimensional Gaussian noises that are independent of each other, and $\Sigma$ is the correlation matrix. In this way, we obtain a micro-state sequence $\boldsymbol{x}_t = (\boldsymbol{S}^{\prime }_t,\boldsymbol{I}^{\prime }_t)$ as the training samples in the experiment. We randomly select initial conditions by sampling points within the triangular region depicted in Fig. 3a and generate time series data using the aforementioned process. These generated data are then utilized to train the models.

We conduct a comparative analysis between NIS+ and other alternative models, including NIS (without EI maximization compared to NIS+), the feed-forward neural network (NN) and the variational autoencoder (VAE). To make a fair comparison, we ensure that all benchmark models have a roughly equal number of parameters. Moreover, we employ the same techniques of probability reweighting and inverse dynamics learning on the feed-forward neural network (NN+) and variational autoencoder (VAE+) as utilized in NIS+. We evaluate the performances of all candidate models by requiring them to predict future states for multiple time steps (10 steps) on a separate test dataset. The results show that NIS+ and NIS outperform other competitors on multi-step prediction, as shown in Fig. 3d, no matter if they use techniques like probability reweighting to maximize EI, which indicates that an invertible neural network as an encoder and decoder is necessary (for details, see Section S5.4).

Furthermore, to assess the model’s generalization ability beyond the region of the training dataset, in addition to the regular training and testing, we also conduct experiments where the model was trained on a subset of the data and tested on the complete dataset. The training samples in this experiment are shown within the dotted area in Fig. 3a (the area with $S \le \frac{1}{3}$ is missing), and the test samples are shown within the triangle. As shown by the red bars in Fig. 3d, the performances of the out-of-distribution generalization of NIS+ are better than other benchmarks, although the test region is beyond the trained region. Also, the differences among different models are larger on the partial dataset.

To further test whether the models successfully learn the ground-truth macro-dynamics, we conduct a comparison between the vector fields of the real SIR dynamics, represented by $\mathrm{d}\boldsymbol{y}/\mathrm{d}t$, and the learned emergent dynamics $\mathrm{d}(h_1,h_2)/\mathrm{d}t$. This comparison is illustrated in Fig. 3c for NIS+ and Fig. 3f for NIS. In both sub-figures, the learned vectors align with the ground-truth (real) dynamics and match the theoretical predictions based on the Jacobian of the encoder (for more details, see Section S5.3). However, it is evident that NIS+ outperforms NIS in accurately capturing the underlying dynamics, especially in peripheral areas with limited training samples.

Next, we test NIS+ and other comparison models on EI maximization and CE quantification; the results are shown in panels (b) and (e) of Fig. 3. First, to ensure that EI is maximized by NIS+, panel (b) illustrates the evolution of EI (dimension averaged) $\mathcal {J}$ over training epochs. It is evident that the curves of NIS+, NIS and VAE+ exhibit upward trends, but NIS+ demonstrates a faster rate of increase. This indicates that NIS+ can efficiently maximize $\mathcal {J}$ to a greater extent than other models. Notably, NIS also exhibits a natural increase in EI as it strives to minimize prediction errors.

Second, to examine NIS+’s ability to detect and quantify CE, we compute the $\Delta \mathcal {J}$ and compare them with $\Psi$ indicators as the noise level $\sigma$ in micro-states increases (see Section S5.2 for details). We utilize the learned macro-states from NIS+ as the prerequisite variable *V* to implement the method in [9]. The results are depicted in Fig. 3e.

Both indicators exhibit a slight increase with $\sigma$ and $\Delta \mathcal {J}> 0$ always holds when it is less than 0.01, but $\Psi > 0$ after $\sigma =10^{-3}$. Therefore, NIS+ indicates that CE consistently occurs at low noise levels, whereas the method in [9] does not. NIS+’s result is more reasonable since it can extract macro-dynamics similar to the ground truth from noisy data, and this deterministic dynamics should have a larger EI than the noisy micro-dynamics. We also plot the curves $J(f_M)$ and $J(f_m)$ for macro- and micro-dynamics, respectively. These curves decrease as $\sigma$ increases, but $J(f_m)$ decreases at a faster rate, leading to the observed occurrence of CE. However, when $\Psi < 0$, we cannot make a definitive judgment as $\Psi$ can only provide a sufficient condition for CE. Both indicators reach their peak at $\sigma =10^{-2}$, which corresponds to the magnitude of the time step ($dt=0.01$) used in our simulations and reflects the level of change in micro-states.

On the other hand, if the noise becomes too large, the limited observational data make it challenging for NIS+ to accurately identify the correct macro-dynamics from the data. Consequently, the degree of CE $\Delta \mathcal {J}$ decreases to zero. Although NIS+ determines that there is no CE when $\sigma > 10$, this result is not reliable since the normalized prediction errors have exceeded the selected threshold 0.3 after $\sigma =10^{-2}$ (the vertical dash–dot line).

These experiments indicate that, by maximizing EI and learning an independent causal mechanism, NIS+ can effectively disregard noise within the data and accurately learn the ground-truth macro-dynamics, as well as generalize to unobservable data. Additionally, NIS+ demonstrates superior performance in quantifying CE. More details regarding the experimental settings are given in Section S5.

### Boids

The second experiment is on the boids model, which is a famous multi-agent model to simulate the collective behaviors of birds [45,46]. In this experiment, we test the ability of NIS+ to capture emergent collective behaviors and CE quantification on different environments with intrinsic and extrinsic noises. To increase the explainability of the trained coarse-graining strategy, we also try to give an explicit correspondence between the learned macro-states and the micro-states.

We simulated the boids model according to the methodology of Reynolds [45] with $N=16$ boids on a $300\times 300$ canvas to generate training data. The detailed dynamical rules of the boids model can be found in Section S6.

**Figure 4.**
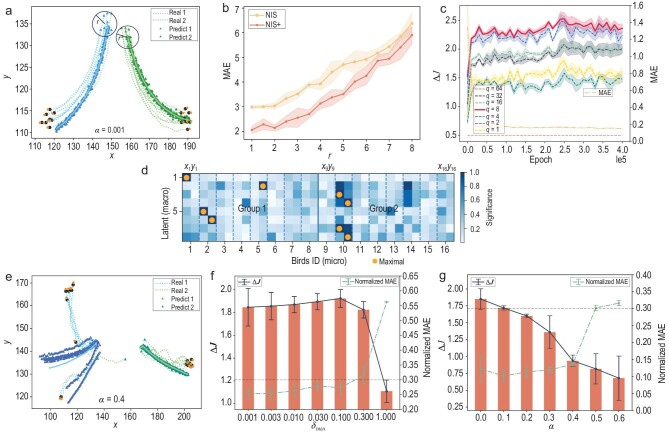
The experimental results of NIS+ on learning the collective flocking behaviors of the boids model. Panels (a) and (e) present real and predicted data on boid trajectories. Concretely, they present the comparison results for multi-step predictions under the condition of two separating groups, and random deflection angles. Panel (b) showcases the escalation of MAE for multi-step predictions as the radius *r*, which represents the range of initial positions of boids in (a), extends beyond the limits of the training data. Panel (c) depicts the trend of $\Delta \mathcal {J}$ changes with training epochs of NIS+ using different hyperparameters of *q*, which represents the scales of different macro-states. Panel (d) presents the saliency map, which visually depicts the association between each macroscopic dimension and the spatial coordinates of each boid. The interpretation can be found in Section S6.2. Panels (f) and (g) show the changes in $\Delta \mathcal {J}$ and normalized MAE under different noise levels for (f) the extrinsic noise ($\delta _{\rm max}$) and (g) intrinsic noise ($\alpha$). In both (f) and (g), the horizontal lines represent the threshold 0.3 for the violation of the constraint of error in Equation (1).

To evaluate the capability of NIS+ in discovering meaningful macro-states, we divided the boids into two groups and introduced distinct constant turning forces for each group. This modification ensured that the two groups followed separate trajectories with different turning angles, as shown in Fig. 4a.

We conducted simulations to generate training and test data for our machine learning model. The micro-state is generated as $4N$-dimensional vectors at each time step *t* as


(6) \begin{eqnarray*}
X_t=\left(x_1^t,y_1^t,v_{x,1}^t,v_{y,1}^t, \dots , x_N^t,y_N^t,v_{x,N}^t,v_{y,N}^t\right),
\end{eqnarray*}

where $(x_i^t,y_i^t)$ is the position and $(v_{x,i}^t,v_{y,i}^t)$ is the velocity at time *t*, $i=1,2,\dots ,16$.

As depicted by the triangles in Fig. 4a, the predicted emergent collective flying behaviors for 50 steps closely follow the ground-truth trajectories of the two groups, particularly at the initial stages. These predicted trajectories are generated by decoding the predicted macro-states into the corresponding micro-states, and the two solid lines represent their averages. The hyperparameter $q=8$, which is the dimension of macro variables, is chosen for this experiment based on the observation that the CE consistently reaches its highest value when $q=8$, as indicated in Fig. 4c.

To enhance the interpretability of the learned macro-states and coarse-graining function in NIS+, we utilize the integrated gradient (IG) method [47] (see Section S3.3) to identify the most significant micro-states for each learned emergent macro-state dimension. We normalized the calculated IG and enhanced the maximum gradient of the micro-state in each macro-state and disregarded the velocity dimensions of each boid due to their lower correlations with macro-states. The matrix diagram of the normalized IG is given in Fig. 4d. As depicted by Fig. 4d, the first, second, fifth and sixth dimensions in macro-states correspond to the boids in the first group (with ${\rm ID}< 8$), while the third, fourth, seventh and eighth dimensions correspond to the second group (with ${\rm ID} \ge 8$). Thus, the learned coarse-graining strategy uses two positional coordinates to represent all other information to form one dimension of the macro-state. For macroscopic states, we need to note that, for a group of birds, two coordinate-related dimensions and two velocity-related dimensions are needed to describe their motion state. So, two groups of birds require eight dimensions. We can speculate that there is one bird as a representative of the group of birds, and observing the situation of that bird can predict the overall movement trend of the group. Then we utilize a doubled number of positional dimensions to make predictions. Aside from the two dimensions representing position, velocity can be derived from the difference in position between two consecutive moments in time. However, only the information from a single moment can be input at a time, necessitating additional degrees of freedom to express velocity. Consequently, we still require eight degrees of freedom to describe the macroscopic state of two groups of birds.

To compare the learning and prediction effects of NIS+ and NIS, we assess their generalization abilities by testing their performances on initial conditions that differed from the training dataset. During the simulation for generating training data, the positions of all boids are constrained within a circle with a radius of *r*, as depicted in Fig. 4a. However, we assess the prediction abilities of both models when the initial positions are located on the larger circles. Figure 4b shows the MAEs of NIS+ and NIS, which increase with the radius *r*, where smaller prediction errors indicate better generalization. The results clearly demonstrate NIS+’s superior generalization across all tested radii *r* compared to NIS.

Furthermore, to examine the impact of intrinsic and observational perturbations on CE, two types of noise are introduced. Intrinsic noise is incorporated into the rule by adding random turning angles to each boid at each time step. These angles are uniformly distributed within the interval $\alpha \cdot [-\pi ,\pi ]$, where $\alpha \in [0,1]$ is a parameter controlling the magnitude of the intrinsic noise. On the other hand, extrinsic noise is assumed to affect the observational micro-states. In this case, we assume that the micro-states of each boid cannot be directly observed, but, instead, noisy data are obtained. The extrinsic or observational noise $\delta \sim \mathcal {N}(0,\delta _{\rm max})$ is added to the micro-states, and $\delta _{\rm max}$ is the parameter determining the level of this noise.

The results are shown in panels (f) and (g) of Fig. 4, where the normalized MAE increases in both cases, indicating more challenging prediction tasks with increasing intrinsic and extrinsic noises. However, the differences between these two types of noise can be observed by examining the degrees of CE ($\Delta \mathcal {J}$). Figure 4f demonstrates that $\Delta \mathcal {J}$ increases with the level of extrinsic noise ($\delta _{\rm max}$), suggesting that coarse graining can mitigate noise within a certain range and enhance causal effects. When $\delta _{\rm max}< 0.1$, the normalized MAE is smaller than 0.3 (dashed horizontal line), satisfying the constraint in Equation (1). In this case, the degree of CE increases with $\delta _{\rm max}$. However, when the threshold of 0.3 is exceeded, and even though $\Delta \mathcal {J}$ decreases, we cannot draw any meaningful conclusion because the violation of the constraint in Equation (1) undermines the reliability of the results.

On the other hand, Fig. 4g demonstrates that $\Delta \mathcal {J}$ decreases as the level of intrinsic noise ($\alpha$) increases. This can be attributed to the fact that the macro-level dynamics learner attempts to capture the flocking behaviors of each group during this stage. However, as the intrinsic noise increases, the flocking behaviors gradually diminish, leading to a decrease in CE. We have not included cases where $\alpha > 0.6$ because the normalized MAE exceeds the threshold of 0.3; the constraints in Equation (1) are violated. Figure 4e illustrates real trajectories and predictions for random deflection angle noise with $\alpha =0.4$. It can be observed that in the early stage, the straight-line trend can be predicted, but, as the noise-induced deviation gradually increases, the error also grows, which intuitively reflects the reduction in CE. To compare, we also test the same curves for $\Psi$; the results are shown in Section S6 because all the values are negative with large magnitudes.

These experiments demonstrate the ability of NIS+ to identify emergent collective behaviors, and how the degree of CE is affected by noise.

### Real fMRI time series data for brains

We test our models on real fMRI time series data of the brains of 830 subjects, called AOMIC ID1000 [48]. The fMRI scanning data are collected when the subjects watch the same movie clip. Thus, similar experiences of subjects under similar natural stimuli are expected, which corresponds to time series of the same dynamics with different initial conditions. The sampling rate (time to repeat) is 2.2 s for ID1000 and 2 s for PIOP2. We pre-process the raw data through the Schaefer atlas method [49] to reduce the dimensionality of the time series for each subject from roughly 140 000 (it varies among subjects) to 100 such that NIS+ can operate and obtain more clear results. Then, the first 800 time series data are selected for training and the remaining 30 time series are for testing. We also compare our results with another fMRI dataset AOMIC PIOP2 [48] for 50 subjects in the resting state. A further description of the dataset can be found in Section S8.

**Figure 5.**
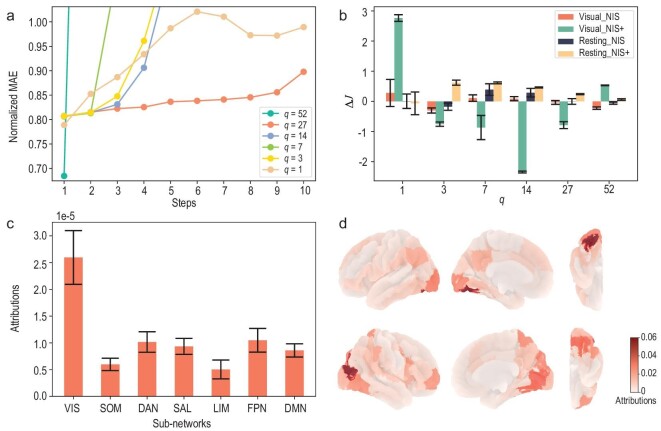
The learning results, the degree of causal emergence and the attribution analysis of NIS+ and NIS on fMRI data of brains. (a) The mean errors of the multi-step predictions increase with the prediction steps under different scales (*q*) on the test dataset. (b) Measures of $\Delta \mathcal {J}$ are compared among different models and different datasets, including movie-watching fMRI (visual fMRI) and resting fMRI. The bars show the averaged results for 10 repeating experiments. The error bars indicate the standard deviation for the last 10 values of CE during the training process. (c) The average attributions of the sub-networks under the Schaefer atlas, calculated using the IG analysis method on the encoder with a scale of $q=1$. The error bars indicate the standard errors for all subjects at different times. (d) Attribution maps for movie-watching (visual) fMRI data. The maps show the left hemisphere from the left, the left hemisphere from the right, the right hemisphere from the right and the right hemisphere from the left. Also, the right column shows a detailed map of the visual areas, with the upper map showing the left visual areas and the lower map showing the right visual areas. The colors represent the normalized absolute values of the integrated gradient.

To demonstrate the predictive capability of NIS+ for micro-states, Fig. 5a illustrates the changes in normalized MAE with the prediction steps of the micro-dynamics on test data for different hyperparameters *q*. It is evident that NIS+ performs better in predictions when $q=27$ and $q=1$. Specifically, the curve for $q=27$ exhibits a slower rate of increase compared to the curve for $q=1$ as the prediction steps increase. This suggests that selecting the hyperparameter *q* as 27 may be more suitable than 1.

However, Fig. 5b suggests a different outcome. When comparing the degree of CE ($\Delta \mathcal {J}$) for different hyperparameters *q* the highest $\Delta \mathcal {J}$ is observed when $q=1$. Conversely, a negative $\Delta \mathcal {J}$ value is obtained when $q=27$. This indicates that the improved prediction results may be attributed to overfitting when $q=27$. Thus, $q=1$ outperforms other values of *q* in terms of $\Delta \mathcal {J}$. This finding is supported by the NIS framework, despite observing a larger standard deviation in $\Delta \mathcal {J}$ when $q=1$. Furthermore, we also compare the results of CE with resting data and observe that peaks are reached at $q=7$, which is just the number of sub-systems in the Schaefer atlas, for both NIS and NIS+. Therefore, we can conclude that, when subjects watch movies, the activities in different brain areas can be represented by a single real number at each time step. More analysis for the resting data is given in Section S8.1. The result is also distinct when applying an alternative framework for identifying causal emergence, as introduced by Rosas *et al.* [9]. For further details, see Sections S1.2 and S8.4. This approach yields exclusively negative values in this experiment, failing to identify causal emergence.

To investigate how NIS+ coarse grains the input data into a single-dimensional macro-state, we also utilize the IG method to identify the most significant dimensions of the micro-state [47]. The results are depicted in panels (c) and (d) of Fig. 5. We observe that the visual (VIS) sub-networks exhibit the highest attribution (Fig. 5c). These visual sub-networks represent the functional system that subjects utilize while watching movie clips. Furthermore, we can visualize the active areas in finer detail on the brain map (Fig. 5d), where darker colors indicate greater attribution to the single macro-state. Therefore, the regions exhibiting similar darkest colors identified by NIS+, which correspond to the deep visual processing brain region, could potentially represent the ‘synergistic core’ [50] when the brain is actively engaged in watching movies. The numeric neurons in these areas may collaborate and function collectively. However, this conclusion should be further confirmed and quantified by decomposing the mutual information between micro-states and macro-states into synergistic, redundant and unique information [9,28].

In conclusion, NIS+ demonstrates its capability to learn and coarse grain the intricate fMRI signals from the brain, allowing for the simulation of complex dynamics using a single macro-state. The robustness of our findings is further supported by comparable results obtained through alternative methods for data pre-processing, as demonstrated in Section S8.

One more experiment is carried out on the classic cellular automata the ‘Game of Life’ that can best exhibit the conception of ‘emergence’. However, due to length constraints, the results are presented in Section S7.

## CONCLUDING REMARKS

Inspired by the theory of causal emergence, this paper introduces a novel machine learning framework called NIS+ to learn emergent macro-dynamics, and suitable coarse-graining methods directly from data. Additionally, it aims to quantify the degree of CE under various conditions.

The distinguishing feature of our framework, compared to other machine learning frameworks, is its focus on maximizing the EI of the learned macro-dynamics while maintaining effectiveness constraints. This enables the learned emergent macro-dynamics to capture the invariant causal mechanism that is as independent as possible from the distribution of input data. This feature not only enables NIS+ to identify CE in data across different environments, but also enhances its ability for generalization on the environments that are distinct from training data. By incorporating the error constraint in Equation (1), we enhance the robustness of the EI maximization framework, addressing the commutativity concerns of renormalization and time evolution operators raised by Eberhardt and Lee [51]. Our framework ensures that micro-dynamics evolution matches the macro-dynamics encoded. As the decoder is the encoder’s inverse, this consistency between evolving micro-states and macro-encoding confirms that the learned dynamics and coarse-graining methods are commutative. As a result, NIS+ extends the theory of CE in Hoel *et al.* [19] to be applicable to both discrete and continuous dynamical systems, as well as real data.

Three experiments were conducted to evaluate the capabilities of NIS+ in learning and generalization, and quantifying CE directly from data. Furthermore, we applied this framework to the domain of the Game of Life (see Section S7). These experiments encompassed three simulation scenarios and one real fMRI dataset for 830 human subjects while watching the same movie clips.

The experiments indicate that, by maximizing EI, NIS+ outperforms other machine learning models in tasks such as multi-step predictions and pattern capturing, even in environments that were not encountered during the training process. Consequently, NIS+ enables the acquisition of a more robust macro-dynamics in the latent space.

Furthermore, the experiments show that NIS+ can quantify CE in a more reasonable way than the $\Psi$ indicator. With this framework, we can distinguish different scenarios in the data and identify which settings contain more regular patterns, as demonstrated in the experiment conducted on the Game of Life. The experiment on the boid model also provides insights into how two types of noise can impact the degrees of CE. The conclusion is that extrinsic noise may increase CE, while intrinsic noise may decrease it. This indicates that extrinsic noise, arising from observational uncertainty, can be mitigated by the learned coarse-graining strategy. On the other hand, intrinsic noise, stemming from inherent uncertainty in the dynamical rules, cannot be eliminated.

NIS+ holds potential for various applications in data-driven modeling of real complex systems, such as climate systems, collective behaviors, fluid dynamics, brain activities and traffic flows. By learning more robust macro-dynamics, the predictive capabilities of these systems can be enhanced. For instance, El Niño, which arises from the intricate interplay of oceanic and atmospheric conditions, exemplifies the emergence of a major climatic pattern from underlying factors. Understanding these emergent macro-dynamics can be instrumental in modeling and predicting El Niño events. By leveraging NIS+ to capture and quantify the CE in such complex systems, we can gain valuable insights and improve our ability to forecast their behavior.

Another interesting merit of NIS+ is its potential contribution to emergence theory by reconciling the debate on whether emergence is an objective concept or an epistemic notion dependent on the observer. By designing a machine to maximize EI, we can extract objective emergent features and dynamics. The machine serves as an observer, but an objective one. Therefore, if the machine observer detects interesting patterns in the data, emergence occurs.

However, there are several limitations in this paper that should be addressed in future studies. Firstly, the requirement of a large amount of training data for NIS+ to learn the macro-dynamics and coarse-graining strategy may not be feasible in many real-world cases. If the training is insufficient, it may lead to incorrect identification of CE. Therefore, it is necessary to incorporate other numeric methods, such as $\Phi$ID [9], to make accurate judgments. One advantage of NIS+ is its ability to identify coarse-grained macro-states, which can then be used as input for the method in [9]. Secondly, the interpretability of neural networks, particularly for the macro-dynamics learner, remains a challenge. Enhancing the interpretability of the learned models can provide valuable insights into the underlying mechanisms and improve the trustworthiness of the results. Thirdly, our work is an extension of the studies presented in [19,20], which assume that the dynamics are Markovian. However, when the dynamics exhibit strong non-Markovian characteristics, alternative frameworks for quantifying emergence, such as those presented in [9,29], may offer superior advantages.

Addressing these limitations and exploring these avenues for improvement will contribute to the advancement of the field and enable the application of NIS+ to a wider range of complex systems.

## METHODS AND DATA

In order to provide a comprehensive understanding of our framework, we introduce why the framework of NIS+ can solve the optimization problem defined in Equation (1). After that, the details of the fMRI time series data are given.

### The model

Solving the optimization problem defined in Equation (1) directly is difficult because the objective function $\mathcal {J}$ is the mutual information after intervention that deserved special process.

To address this challenge, we transform the issue as delineated in Equation (1) into a new optimization problem without constraints, that is,


(7) \begin{eqnarray*}
&&\min _{f,g,\phi ,\phi ^{\dagger }} \sum _{t=1}^{T-1} w(\boldsymbol{x}_t)||\boldsymbol{y}_t-g(\boldsymbol{y}_{t+1})||\\
&&\quad +\, \lambda || \hat{\boldsymbol{x}}_{t+1}-\boldsymbol{x}_{t+1} ||,
\end{eqnarray*}

where $\hat{\boldsymbol{x}}_{t+1}=\phi ^{\dagger }(f(\phi (\boldsymbol{x}_t)))$; $\boldsymbol{y}_t=\phi (\boldsymbol{x}_t)$ and $\boldsymbol{y}_{t+1}=\phi (\boldsymbol{x}_{t+1})$ are the macro-states; $g:\mathcal {R}^q\rightarrow \mathcal {R}^q$ is a new function that we introduce to simulate the inverse macro-dynamics on the macro-state space, that is, to map each macro-state at the $t+1$ time step back to the macro-state at the *t* time step. The parameter $\lambda$ serves as a Lagrangian multiplier, to be considered a tunable hyperparameter within our experimental framework. The inverse probability weights, denoted $w(\boldsymbol{x}_t)$, are characterized by the definition


(8) \begin{eqnarray*}
w(\boldsymbol{x}_t)=\frac{\tilde{p}(\boldsymbol{y}_t)}{p(\boldsymbol{y}_t)} =\frac{\tilde{p}(\phi (\boldsymbol{x}_t))}{p(\phi (\boldsymbol{x}_t))}.
\end{eqnarray*}

In this context, $\tilde{p}$ represents the modified distribution of macro-states $\boldsymbol{y}_t$ following the intervention according to ${\rm do}(\boldsymbol{y}_t\sim U_q)$, whereas *p* denotes the inherent distribution of the observed data. For practical implementation, $p(\boldsymbol{y}_t)$ is approximated using kernel density estimation [52] (further elaborated in Section S3.2). The post-intervention distribution $\tilde{p}(\boldsymbol{y}_t)$ is presumed to be uniformly distributed, signified by a consistent value across its range. As a result, the weight *w* is determined by the ratio of the original to the modified distribution. Mathematical theorems mentioned in Section S2.2 and proven in Section S4.2 guarantee that this new optimization problem (Equation (7)) is equivalent to the original one (Equation (1)).

### fMRI time series data

AOMIC is an fMRI collection that comprises AOMIC PIOP1, AOMIC PIOP2 and AOMIC ID1000 [48].

AOMIC PIOP2 collected subjects’ data for multiple tasks such as emotion matching, working memory and so on (${\rm TR}=2$ s). Here, we just use 50 subjects’ resting fMRI data since some time steps of other subjects have been thrown out by removing the effects of artificial motion, the global signal, the white matter signal and the cerebrospinal fluid signal using fMRIPrep results [53,54], which leads to a difficulty in time alignment.

AOMIC ID 1000 collected data when 881 subjects were watching movies. It contains both raw data and pre-processed data (${\rm TR}=2.2$ s; see further experimental and pre-processing details in [48]). Here, we should note that the movie is edited in such a way that it has no clear semantic meaning, but is just a collection of concatenated images from the movie. Therefore, it is expected that subjects’ brain activation patterns should not respond to some higher-order functions such as semantic understanding. The detailed pre-process method is presented in Section S8.3.

## Supplementary Material

nwae279_Supplemental_File

## Data Availability

All the codes and data are available at https://github.com/Matthew-ymz/Code-for-Finding-emergence-in-data-5.2.
