# Supplementary material for: Finding emergence in data by maximizing effective information
Source: Natl Sci Rev. 2024 Aug 12;12(1):nwae279. doi: 10.1093/nsr/nwae279 (PMC11697982; doi:10.1093/nsr/nwae279)
Supplement: nwae279_Supplemental_File [file nwae279_supplemental_file.pdf]

Support Information

Contents

1 A Brief Introduction of Causal Emergence Theory **3**

1.1 Erik Hoel's Causal Emergence Theory . . . 3

1.2 Fernando E. Rosas's Quantification of Causal Emergence . . . 3

2 The Frameworks and Mathematical Thoerems of NIS and NIS+ **4**

2.1 Neural Information Squeezer (NIS) . . . 4

2.2 Neural Information Squeezer Plus (NIS+) . . . 5

2.3 Invertible neural network in encoder(decoder) . . . 6

2.4 Extensions for Practical Computations . . . 7

2.5 Training NIS+ . . . 9

3 Miscellaneous **9**

3.1 Measures and calculations of EI and causal emergence for neural networks . . . 9

3.2 KDE for probability reweighting . . . 10

3.3 Integrated Gradients for attribution . . . 11

4 Mathematical Proves **11**

4.1 Preceding instructions . . . 11

4.2 Proof of Theorem 2.1 . . . 11

4.3 Proof of Theorem 2.2 . . . 15

4.4 Proof of Theorem 2.3 . . . 16

5 Details about the experiments in SIR Model **18**

5.1 Data generation . . . 18

5.2 Training and testing details . . . 18

5.3 Methods for the vector field results . . . 19

5.4 Comparison models in the SIR experiment . . . 21

6 Details in the experiments of Boids Model **21**

6.1 Method of Simulating the Trajectory of Boids . . . 21

6.2 Methods for Collecting Experimental Data and Training . . . 23

7 Game of Life **24**

7.1 Data generation . . . 25

7.2 The model predictive ability on glider pattern 27

8 Details about the experiments in fMRI 27

8.1 About resting state 27

8.2 AAL Results 27

8.3 Pre-processing fMRI time series data in detail 27

8.4 Compare with other framework for identifying causal emergence 28

9 The Structure and parameters of neural networks. 28

1. A Brief Introduction of Causal Emergence Theory

1.1. Erik Hoel's Causal Emergence Theory

The basic idea of [1] is that we have two different views (micro and macro) of a same Markov dynamical system, and CE occurs if the macro-dynamics has stronger causal effect than the micro-dynamics. As shown in Figure 1 of the main text, for example, the micro-scale system is composed by many colliding balls. The macro-scale system is a coarse-grained description of the colliding balls with colorful boxes where each box represents the number of balls within the box. In Figure 1 of the main text, the vertical axis represents the scale(micro- and macro-), and the horizontal axis represents time, which depicts the evolution of the system's dynamics in both scales. All the dynamical systems considered are Markovian, therefore two temporal successive snapshots are necessary. The dynamics in both micro- and macro-scales are represented by transitional probability matrix(TPM): f_m for micro-dynamics and f_M for macro-dynamics.

We can measure the strength of its causal effects for each dynamics(TPM) by effective information (EI). EI can be understood as an intervention-based version of mutual information between two successive states in a dynamical system over time. For any TPM f, EI can be calculated by:

$$\mathcal{J} = I(S_t; S_{t-1}|do(S_{t-1} \sim U(S))) = \frac{1}{N}\sum_{i,j=1}^{N}\left(f_{ij}\log f_{ij} - f_{ij}\log\left(\frac{\sum_{k=1}^{N} f_{kj}}{N}\right)\right), \quad (1)$$

where, S_t is the random variable to represent the state of the system at time t, $do(S_{t-1} \sim U(S))$ represents the do-operator [2] that intervene the state of the system at time $t-1$ to force that S_{t-1} follows a uniform distribution on the value space S of S_t. f_{ij} is the probability at the ith row and the jth column. $N = |S|$ is the total number of states and is also the number of rows or columns. log represents the logarithmic operation with a base of 2. Equation 1 can be further decomposed into two terms: determinism and non-degeneracy which characterize that how the future state can be determined by the past state and vice versa, respectively[1].

With EI, we can compare two TPMs. If $\mathcal{J}(f_M)$ is larger than $\mathcal{J}(f_m)$, we say CE occurs in this dynamical system. In practice, we calculate another value called causal emergence to judge if CE takes place, that is:

$$\Delta\mathcal{J} = \mathcal{J}(f_M) - \mathcal{J}(f_m). \quad (2)$$

Therefore, if $\Delta\mathcal{J} > 0$, then CE occurs. $\Delta\mathcal{J}$ can be treated as the quantitative measure of emergence for Markov dynamics. An example of CE on Markov chain is shown in Figure 1(b) of the main text.

1.2. Fernando E. Rosas's Quantification of Causal Emergence

To circumvent the necessity of manually selecting the coarse-graining function and intervening in the data, which are limitations inherent to Erik Hoel's causal emergence theory, Rosas et al. have introduced a novel approach. This approach involves decomposing the excess entropy—the mutual information between the system's past and future states—to quantify causal emergence. They have employed Partial Information Decomposition (PID) [3] to partition the excess entropy into distinct components and identify those parts that are pertinent to the phenomenon of emergence.

Suppose there are three variables X_1, X_2 and Y. We can decompose the mutual information $I(X_1, X_2; Y)$ into three types of information: Redundant information(Red), which refers to the information held by both sources ; Unique information(Un), which refers to the information held by one source but not the other; Synergy information(Syn), which is the information held by all sources together, but not by any individual source.

$$I(X_1, X_2; Y) = Red(X_1, X_2; Y) + Un(X_1; Y|X_2) + Un(X_2; Y|X_1) + Syn(X_1, X_2; Y) \quad (3)$$

However, PID can only be employed on a single target variable, which means it cannot be used for excess entropy. To address this issue, Rosas et al. proposed a ϕID framework to extend PID for the decomposition of excess entropy [4]. They decomposed the joint mutual information into several non-overlapping information atoms $I_\partial^{\alpha\to\beta}$, that is:

$$\mathbf{E} = I(\mathbf{X}_t; \mathbf{X}_{t+1}) = \sum_{\alpha,\beta\in\mathcal{A}} I_\partial^{\alpha\to\beta} \tag{4}$$

These information atoms represent, respectively, the redundant information, synergistic information, and unique information between multiple variables from time t to $t+1$. Causal emergence can be understood as the synergistic characteristics of a system's micro-level variables; therefore, synergistic information can be used to determine whether causal emergence has occurred [5]:

$$Syn(X_t; X_{t+1}) > 0. \tag{5}$$

However, calculating information atoms presents a significant challenge. Consequently, the author aims to identify the lower bounds of the original definition to establish more computationally convenient sufficient conditions. If a specific macroscopic variable V can be identified through a particular function mapping, the unique information that macroscopic variables hold about microscopic variables could serve as a sufficient condition for causal emergence:

$$Syn(X_t; X_{t+1}) \geq Un(V_t; X_{t+1}|X_t) > 0. \tag{6}$$

Furthermore, the calculation of unique information can be superseded by employing the formula for mutual information:

$$Un(V_t; X_{t+1}|X_t) \geq I(V_t; V_{t+1}) - \sum_j I(X_t^j; V_{t+1}) + Red(V_t, V_{t+1}; X_t). \tag{7}$$

Since redundant information cannot be calculated, a sufficient condition Ψ has been established to replace the original measurement method:

$$\Psi_{t,t+1}(V) := I(V_t; V_{t+1}) - \sum_j I(X_t^j; V_{t+1}) > 0. \tag{8}$$

In the given context, X_t^j denotes the microscopic variable of the j-th dimension. The Ψ mentioned here refers to the Ψ calculated in the main body of the experiment. According to the theory in [5], Ψ being greater than 0 is a sufficient but not necessary condition for emergence to occur. As we can observe, the measure Ψ differs from the original definition of causal emergence by at least a redundant information. Consequently, in systems with numerous variables, redundant information dominates, rendering Ψ ineffective for identifying causal emergence. In this paper, we employ the method proposed by Lu et al. [6] to compute the continuous variable mutual information in the experimental configuration.

2. The Frameworks and Mathematical Thoerems of NIS and NIS+

We will introduce the details of the two frameworks NIS and NIS+ in this section.

2.1. Neural Information Squeezer (NIS)

NIS use neural networks to parameterize all the functions to be optimized in Equation 1 of the main text, in which the coarse-graining and anti-coarsening function ϕ and $\phi^\dagger$ are called encoder and decoder, respectively, and the macro-dynamics f_q is called dynamics-learner. Second, considering the symmetric position between ϕ and $\phi^\dagger$, invertible neural network(with RealNVP framework [7], details can be referred to the following sub-section 2.3) is used to reduce the model complexity and to make mathematical analysis being possible [8].

Concretely,

$$\phi \equiv Proj_q(\psi_\omega), \tag{9}$$

where $\psi_\omega : \mathcal{R}^p \to \mathcal{R}^p$ is an invertible neural network with parameters ω, and $Proj_q$ represents the projection operation with the first q dimensions reserved to form the macro-state $\boldsymbol{y}$, and the last $p-q$ dimensional variable $\boldsymbol{y}'$ are dropped. Empirically, $\boldsymbol{y}'$ can be approximately treated as a Gaussian noise and independent with $\boldsymbol{y}$ or we can force $\boldsymbol{y}'$ to be independent Gaussian by training the neural network. Similarly, in a symmetric way, $\phi^\dagger$ can be approximated in the following way: for any input $\boldsymbol{y} \in \mathcal{R}^q$,

$$\phi^\dagger(\boldsymbol{y}) = \psi_\omega^{-1}(\boldsymbol{y} \bigoplus \xi), \tag{10}$$

where ξ is a standard Gaussian random vector with $p-q$ dimensions, and $\bigoplus$ represents the vector concatenation operation.

Last, the macro-dynamics f_q can be parameterized by a common feed-forward neural network f_θ with weight parameters θ. Its number of input and output layer neurons equal to the dimensionality of the macro state q. It has two hidden layers, each with 64 neurons, and the output is transformed using LeakyReLU. The detailed architectures and parameter settings of ψ_ω and f_θ can be referred to the support information section 9. To compute EI, this feed-forward neural network is regarded as a Gaussian distribution, which models the conditional probability $p(\hat{\boldsymbol{y}}_{t+1}|\boldsymbol{y}_t)$.

Previous work [8] has been proved that this framework merits many mathematical properties such as the macro-dynamics f_θ forms an information bottleneck, and the mutual information between $\boldsymbol{y}_t$ and $\hat{\boldsymbol{y}}_{t+1}$ approximates the mutual information between $\boldsymbol{x}_t$ and $\boldsymbol{x}_{t+1}$ when the neural networks are convergent such that the constraint in Equation 1 of the main text is required.

2.2. Neural Information Squeezer Plus (NIS+)

To maximize EI defined in Equation 1 of the main text, we extend the framework of NIS to form NIS+. In NIS+, we first use the formula of mutual information and variational inequality to convert the maximization problem of mutual information into a machine learning problem, and secondly, we introduce a neural network $g_{\theta'}$ to learn the inverse macro-dynamics that is to use $\boldsymbol{y}_{t+1} = \phi(\boldsymbol{x}_{t+1})$ to predict $\boldsymbol{y}_t$ such that mutual information maximization can be guaranteed. Third, the probability re-weighting technique is employed to address the challenge of computing intervention for a uniform distribution, thereby optimizing the EI. All of these techniques compose neural information squeezer plus (NIS+).

Formally, the maximization problem under the constraint of inequality defined by Equation 1 of the main text can be converted as a minimization of loss function problem without constraints, that is:

$$\min_{\omega,\theta,\theta'} \sum_{t=1}^{T-1} w(\boldsymbol{x}_t)||\boldsymbol{y}_t - g_{\theta'}(\boldsymbol{y}_{t+1})|| + \lambda||\hat{\boldsymbol{x}}_{t+1} - \boldsymbol{x}_{t+1}||, \tag{11}$$

where ω, θ, θ' are the parameters of neural networks of ψ_ω, f_θ, and $g_{\theta'}$, respectively. $\boldsymbol{y}_t = \phi(\boldsymbol{x}_t) = Proj_q(\psi_\omega(\boldsymbol{x}_t))$ and $\boldsymbol{y}_{t+1} = \phi(\boldsymbol{x}_{t+1}) = Proj_q(\psi_\omega(\boldsymbol{x}_{t+1}))$ are the macro-states. λ is a Lagrangian multiplier which will be taken as a hyper-parameter in experiments. $w(\boldsymbol{x}_t)$ is the inverse probability weights which is defined as:

$$w(\boldsymbol{x}_t) = \frac{\tilde{p}(\boldsymbol{y}_t)}{p(\boldsymbol{y}_t)} = \frac{\tilde{p}(\phi(\boldsymbol{x}_t))}{p(\phi(\boldsymbol{x}_t))}, \tag{12}$$

where $\tilde{p}$ is the new distribution of macro-states $\boldsymbol{y}_t$ after intervention for $do(\boldsymbol{y}_t \sim U_q)$, and p is the natural distribution of the data. In practice, $p(\boldsymbol{y}_t)$ is estimated through kernel density estimation (KDE)[9](Please refer the support information section 3.2). The approximated distribution, $\tilde{p}(\boldsymbol{y}_t)$, is assumed to be a uniform distribution, which is characterized by a constant value. Consequently, the weight w is computed as the ratio of these two distributions.

We can prove a mathematical theorem to guarantee such problem transformation as mentioned below:

Theorem 2.1 (Problem Transformation Theorem)**.** *For a given value of q, the unconstrained objective function outlined by Eq.(11) serves as the lower bound of constrained objective function defined by Equation 1 of the main text.*

The details of proof can be seen in support information section 4.2.

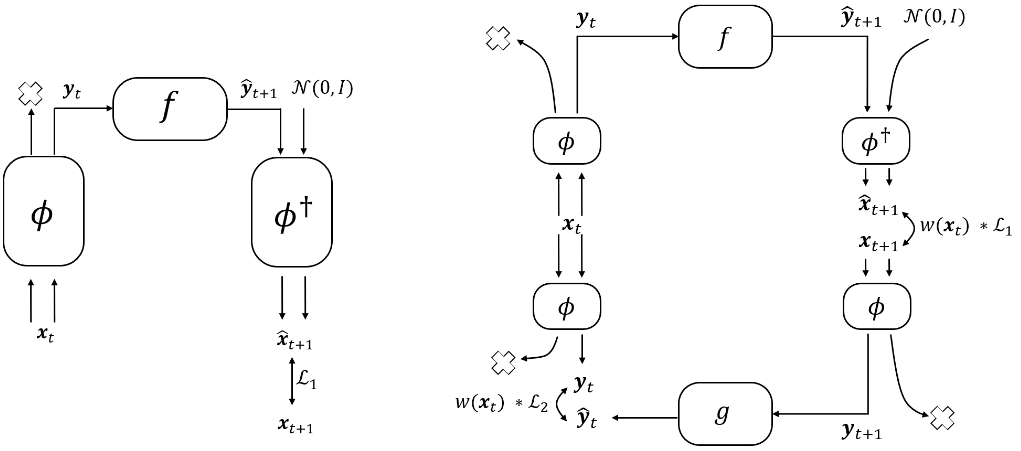

(a). NIS (b). NIS+

Figure 1. The frameworks of NIS (a) and NIS+ (b). The boxes in the diagram represent functions or neural networks, while the arrow pointing to a cross signifies the operation of information dropping. $\boldsymbol{x}_t$ and $\boldsymbol{x}_{t+1}$ represent the observational data of micro-states, and $\hat{\boldsymbol{x}}_{t+1}$ represents the predicted micro-state. The macro-states, denoted as $\boldsymbol{y}_t = \phi(\boldsymbol{x}_t)$ and $\boldsymbol{y}_{t+1} = \phi(\boldsymbol{x}_{t+1})$, are obtained by encoding the micro-states using the encoder. Similarly, the predicted macro-states, $\hat{\boldsymbol{y}}_t = \phi(\hat{\boldsymbol{x}}_t)$ and $\hat{\boldsymbol{y}}_{t+1} = \phi(\hat{\boldsymbol{x}}_{t+1})$, are obtained by encoding the predictions of micro-states.

Discriminate from Figure 1(a), a new computational graph for NIS+ which is depicted in Figure 1(b) is implied by the optimization problem defined in Equation 11. For given pair of data: $\boldsymbol{x}_t, \boldsymbol{x}_{t+1}$, two dynamics, namely the forward dynamics f_θ and the reverse dynamics $g_{\theta'}$ are trained by optimizing two objective functions $\mathcal{L}_1 = \sum_{t=1}^{T-1} w(\boldsymbol{x}_t)||\boldsymbol{y}_t - g_{\theta'}(\boldsymbol{y}_{t+1})||$ and $\mathcal{L}_2 = \sum_{t=1}^{T-1} ||\hat{\boldsymbol{x}}_{t+1} - \boldsymbol{x}_{t+1}||$, simultaneously. In the training process, the encoder, ϕ and the decoder, $\phi^\dagger$ are shared.

This novel computational framework of NIS+ can realize the maximization of EI under the coarse-grained emergent space. Therefore, it can optimize an independent causal mechanism(f) represented by f_θ on the emergent space. It can also be used to quantify CE in raw data once the macro-dynamics f_θ is obtained for different q.

2.3. Invertible neural network in encoder(decoder)

In NIS, both the encoder(ϕ) and the decoder($\phi^\dagger$) use the invertible neural network module RealNVP[7].

Concretely, if the input of the module is $\mathbf{x}$ with dimension p and the output is $\mathbf{x}'$ with the same dimension, then the RealNVP module can perform the following computation steps:

$$\begin{cases} \mathbf{x_1} = \mathbf{x}_{1:m} \\ \mathbf{x_2} = \mathbf{x}_{m:p} \end{cases} \tag{13}$$

where, m is an integer in between 1 and p.

$$\begin{cases} \mathbf{x}'_1 = \mathbf{x_1} \bigotimes s_1(\mathbf{x_2}) + t_1(\mathbf{x_2}) \\ \mathbf{x}'_2 = \mathbf{x_2} \bigotimes s_2(\mathbf{x}'_1) + t_2(\mathbf{x}'_1) \end{cases} \tag{14}$$

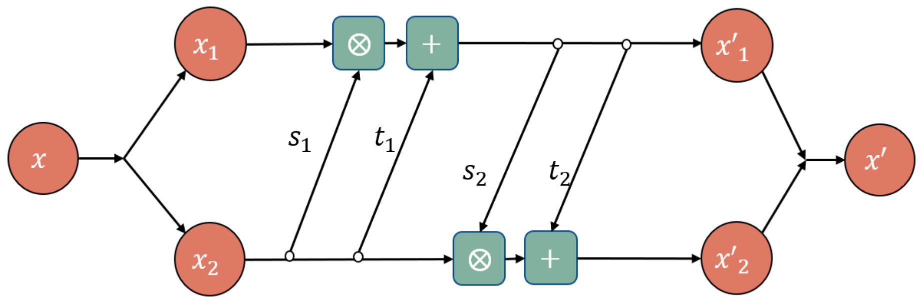

Figure 2. Structure diagram of RealNVP.

where, $\bigotimes$ is element-wised time, s_1, s_2, t_1 and t_2 are feed-forward neural networks with arbitrary architectures, while their input-output dimensions must match with the data. For the experiments in this article, they each have two intermediate hidden layers. The input and output layers have the same number of neurons as the dimensions of the micro-state samples. Each hidden layer has 64 neurons, and the output of each hidden layer is transformed by the non-linear activation function LeakyReLU. In practice, s_1 or s_2 always do an exponential operation on the output of the feed-forward neural network to facilitate the inverse computation.

Finally,

$$\mathbf{x}' = \mathbf{x}_1' \bigoplus \mathbf{x}_2' \tag{15}$$

It is not difficult to verify that all three steps are invertible. Equation 14 is invertible because the same form but with negative signs can be obtained by solving the expressions of $\mathbf{x_1}$ and $\mathbf{x_2}$ with $\mathbf{x}_1'$ and $\mathbf{x}_2'$ from Equation 14.

To simulate more complex invertible functions, we always duplex the basic RealNVP modules by stacking them together. In the main text, we use duplex the basic RealNVP module by three times.

Due to the reversibility of RealNVP, if its output is used as input in a subsequent iteration, the new output will be the same as the original input. Therefore, the neural networks used in the encoder-decoder can share parameters. The process of encoding to obtain the macro-state involves projecting the forward output and retaining only the first few dimensions. On the other hand, the process of decoding to obtain the micro-state involves concatenating the macro-state with a normal distribution noise, expanding it to match the micro-state dimensions, and then feeding it back into the entire invertible neural network.

2.4. Extensions for Practical Computations

When we apply NIS+ to the data generated by various complex systems such as cellular automata and multi-agent systems, the architecture of encoder(decoder) should be extended for stacked and parallel structures. Fortunately, NIS+ is very flexible to different real scenarios which means the important properties such as Theorem 2.1 can be retained.

Firstly, it is important to consider that when dealing with high-dimensional complex systems, discarding multiple dimensions at once can pose challenges for training neural networks. Therefore, we replace the encoder in NIS+ with a **Stacked Encoder**. As shown in Figure 3(a), this improvement involves stacking a series of basic encoders together and gradually discard dimensions, thereby reducing the training difficulty.

In addition, complex systems like cellular automota are always comprise with multiple components with similar dynamical rules. Therefore, we introduce the **Parallel Encoder** to represent each component or groups of these components and combine the results together. Concretely, as shown in Figure 3(b), inputs may be grouped based on their physical relationships(e.g. adjacent neighborhood), and each group is encoded using a basic encoder. By sharing parameters among the encoders, neural networks can capture homogeneous coarse-graining rules efficiently and accurately. Finally, the macro variables obtained from all the encoders are concatenated into a vector

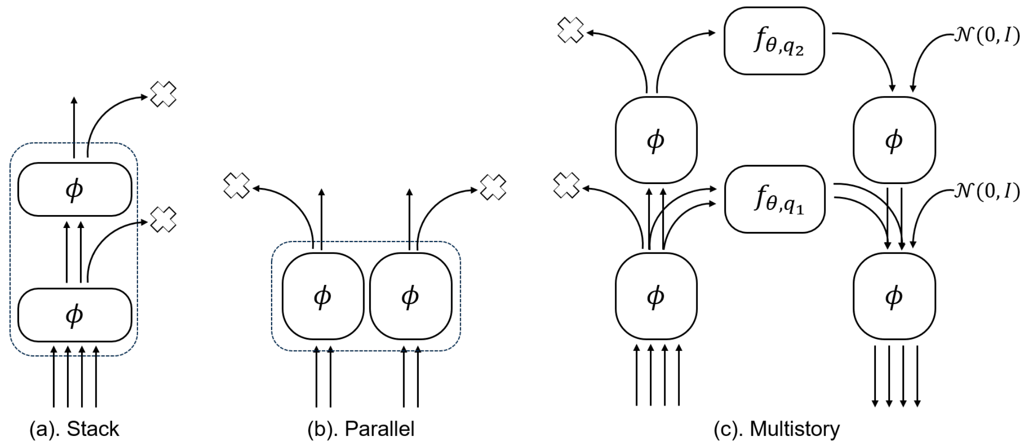

Figure 3. Three extended structures of the NIS+ encoder, denoted as (a) and (b), as well as the complete structure (c), are designed to facilitate the implementation of complex tasks. (a) represents a stacked encoder, where basic encoders are layered one on top of another. This arrangement allows for the filtering of input information in a hierarchical manner. (b) showcases a parallel encoder, where multiple basic encoders with shared parameters are combined to form a larger encoder. This parallel configuration enables the processing of input information in parallel, enhancing efficiency. (c) illustrates a multistory structure of the complete NIS+ framework. In this structure, the encoders and decoders are stacked, and the dynamics learners in different layers operate in parallel. This design facilitates simultaneous training for different dynamics learners with distinct dimensions and enables parallel searching for the optimal dimension q.

to derive the overall macro variables. Furthermore, we can combine this parallel structures with other well-known architectures like convolutional neural networks.

To enhance the efficiency of searching for the optimal scale, we leverage the multiple scales of hidden space obtained through the stacked encoder by training multiple dynamics learners in different scales simultaneously. This forms the framework of **Multistory NIS+** as shown in Figure 3(c). This approach is equivalent to searching macro-dynamics for different q and thereby avoiding to retrain the encoders.

Theorem 2.2 guarantees that the important properties such as Theorem 2.1 can be retained for the extensions of stacked and parallel encoders. And a new universal approximation theorem (Theorem 2.3) can be proved such that multiple stacked encoders can approximate any coarse-graining function (any map defined on $\mathcal{R}^p \times \mathcal{R}^q$).

Theorem 2.2 (Problem Transformation in Extensions of NIS+ Theorem)**.** *When the encoder of NIS+ is replaced with an arbitrary combination of stacked encoders and parallel encoders, the conclusion of Theorem 2.1 still holds true.*

Theorem 2.3 (Universal Approximating Theorem of Stacked Encoder)**.** *For any continuous function f which is defined on $K \times \mathcal{R}^p$, where $K \in \mathcal{R}^p$ is a compact set, and $p > q \in \mathcal{Z}^+$, there exists an integer s and an extended stacked encoder $\phi_{p,s,q} : \mathcal{R}^p \to \mathcal{R}^q$ with s hidden size and an expansion operator $\eta_{p,s}$ such that:*

$$\phi_{p,s,q} \simeq f, \tag{16}$$

Thereafter, an extended stacked encoder with expansion operators is of universal approximation property which means that it can approximate and simulate any coarse-graining function defined on $\mathcal{R}^p \times \mathcal{R}^q$.

The extended stacked encoder $\phi_{p,s,q}$ refers to stacking two basic encoders together and gradually reducing dimensions, encoding the input from p dimensions to q dimensions, with an intermediate

dimension of s. Besides basic operations such as invertible mapping and projection, a new operation, vector expansion($\eta_{p,s}$, e.g., $\eta_{2,5}(1,2,3) = (1,2,3,1,2)$), should be introduced, and locate in between the two encoders. All the proves of these theorems are referred to support information section 4.3 and 4.4.

2.5. Training NIS+

In practice, two stages are separated during training process for NIS+ and all extensions. The first stage only trains the forward neural networks (the upper information channel as shown in Figure 2 of the main text) such that the loss $\mathcal{L}_1$ is small enough. Then, the second stage which only trains the neural networks for the reverse dynamics (the lower information channel as shown in Figure 2 of the main text) is conducted. Because the trained inverse dynamics $g_{\theta'}$ is never used, the second stage is for training ϕ_ω in essence.

3. Miscellaneous

3.1. Measures and calculations of EI and causal emergence for neural networks

The measure of EI of discrete Markov chains can be calculated by Equation 1, however, it is not suitable for neural networks because a neural network is a deterministic function. Therefore, we need to extend the definition of EI for general neural networks. We continue to use the method defined in [8]. The basic idea is to understand a neural network as a Gaussian distribution conditional on the input vector with the mean as the deterministic function of the input values, and the standard deviations as the mean square errors of this neural network on the training or test datasets.

In general, if the input of a neural network is $X = (x_1, x_2, \cdots, x_n) \in [-L, L]^n$, which means X is defined on a hyper-cube with size L, where L is a very large integer. The output is $Y = (y_1, y_2, \cdots, y_m)$, and $Y = \mu(X)$. Here μ is the deterministic mapping implemented by the neural network: $\mu : \mathcal{R}^n \to \mathcal{R}^m$, and its Jacobian matrix at X is $\partial_{X'}\mu(X) \equiv \left\{ \frac{\partial \mu_i(X')}{\partial X'_j} |_{X'=X} \right\}_{nm}$. If the neural network can be regarded as a Gaussian distribution conditional on given X, then the effective information (EI) of the neural network can be calculated in the following way:

$$EI_L(\mu) = I(do(X \sim U([-L,L]^n; Y) \approx -\frac{m + m\ln(2\pi) + \sum_{i=1}^m \sigma_i^2}{2} + n\ln(2L) + \mathbf{E}_{X\sim U([-L,L]^n} \left(\ln|\det(\partial_{X'}\mu(X))|\right). \tag{17}$$

where, $\Sigma = diag(\sigma_1^2, \sigma_2^2, \cdots, \sigma_m^2)$ is the co-variance matrix, and σ_i is the standard deviation of the output y_i which can be estimated by the mean square error of y_i under given x_i. $U([-L, L]^n)$ is the uniform distribution on $[-L, L]^n$, and $|\cdot|$ is absolute value, and det is determinant. If $\det(\partial_{X'}\mu(X)) \equiv 0$ for all X, then we set $EI \approx 0$.

However, Equation 17 can not be applied in real cases directly because it will increase as the dimension of input n or output m increases[8]. Therefore, larger EI is always expected for the models with higher dimension. The way to solve this problem is by dividing the input dimension to define dimension averaged effective information(dEI) and is denoted as $\mathcal{J}$:

$$\mathcal{J}_L = \frac{EI_L(\mu)}{n} \tag{18}$$

When the numbers of input and output are identical ($m = n$, this condition is always hold for all the results reported in the main text), Equation 17 becomes:

$$\mathcal{J}_L(\mu) = -\frac{1 + \ln(2\pi) + \sum_{i=1}^n \sigma_i^2/n}{2} + \ln(2L) + \frac{1}{n}\mathbf{E}_{X\sim U([-L,L]^n} \left(\ln|\det(\partial_{X'}f(X))|\right). \tag{19}$$

We always use this measure to quantify EI for neural networks in the main text. However, this measure is also not perfect because it is dependent on a free parameter L, the range of the

domain for the input data. Our solution is to calculate the dimension averaged CE to eliminate the influence of L. For macro-dynamics f_M with dimension q and micro-dynamics f_m with dimension p, we define dimension averaged CE as:

$$\Delta\mathcal{J}_L(f_M, f_m) = \mathcal{J}_L(f_M) - \mathcal{J}_L(f_m) = \frac{EI_L(f_M)}{q} - \frac{EI_L(f_m)}{p}. \tag{20}$$

If f_M and f_m are parameterized by neural networks of μ_M with dimension q and μ_m with dimension p, then

$$\begin{aligned}\Delta\mathcal{J} = &\left(\frac{1}{q}\mathbf{E}_{X_M} \ln|\det \partial_{X_M}\mu_M| - \frac{1}{p}\mathbf{E}_{X_m} \ln|\det \partial_{X_m}\mu_m|\right) \\ &- \left(\frac{1}{q}\sum_{i=1}^{q} \ln\sigma_{i,M}^2 - \frac{1}{p}\sum_{i=1}^{p} \ln\sigma_{i,m}^2\right),\end{aligned} \tag{21}$$

where $\sigma_{i,M}$ and $\sigma_{i,m}$ are the standard deviations for μ_M and μ_m on the ith dimension, respectively. At this point, the influences of the input or output dimensions and the parameter L has been completely eliminated, making it a more reliable indicator. Therefore, the comparisons between the learned macro-dynamics reported in the main text are reliable because the measure of dimension averaged CE is used.

In practice, the first step is to select a sufficiently large value for L to ensure that all macro-states are encompassed within the region $[-L, L]^q$. Subsequently, the determinant of Jacobian matrices can be estimated using Monte Carlo integration, which involves sampling data points within this region. The standard deviations can be estimated using a test dataset, and the probability reweighting technique is employed to account for interventions and ensure a uniform distribution.

3.2. KDE for probability reweighting

In order to use inverse probability weighting technique, we need to estimate the probability distribution of the samples. KDE(Kernel Density Estimation) is a commonly used estimation method that can effectively eliminate the influence of outliers on the overall probability distribution estimation. In this experiment, we estimate the probability distribution of macro-states $\boldsymbol{y}_t$. Below is an introduction to the operation details of KDE. For a sample $(x_1, x_2, \cdots, x_n)$, we have the following kernel density estimation:

$$\hat{f}_h(x) = \frac{1}{nh}\sum_{i=1}^{n} K(\frac{x - x_i}{h}). \tag{22}$$

n represents the number of samples, the hyperparameter h denotes the bandwidth, which is determined based on the rough range of the data and typically set to 0.05 in this article. K is the kernel, specifically the standard normal density function. After obtaining the estimation function, each sample point is evaluated individually, resulting in the corresponding probability value for each sample point. By dividing the probability of the target distribution (uniform distribution) by our estimated probability value, we obtained the inverse probability weights corresponding to each sample point. To cover all sample points, the range of the uniform distribution needs to be limited by a parameter L, which ensures that a square with side length $2L$ can encompass all the sample points in all dimensions.

Another thing to note is that the weights obtained at this point are related to the sample size, so they need to be normalized. However, this will result in very small weight values corresponding to the samples. Therefore, it is necessary to multiply them by the sample quantity to amplify the weight values back to their normal scale. Then, multiply this weight value by the training loss of each sample to enhance the training of sparse regions, achieving the purpose of inverse probability weighting. Since the encoder parameters change with each iteration, causing the distribution of $\boldsymbol{y}_t$ to change, we will re-estimate the probability distribution of the entire sample using KDE every 1000 epochs (a total of at least 30000 epochs of iteration, the number of iterations may vary in different experiments).

3.3. Integrated Gradients for attribution

Here we introduce a method called Integrated Gradients[10] that is used to explain the connection between macro-states and micro-states. To formally describe this technique, let us consider a function $F : \mathcal{R}^p \to [0,1]$ that represents a deep network. Specifically, let $x \in \mathcal{R}^p$ denote the input under consideration, and $x' \in \mathcal{R}^p$ denote the baseline input. In both the fMRI and Boids experiments, the baseline is set to 0.

We compute the gradients at each point along a straight-line path (in $\mathcal{R}^p$) from the baseline x' to the input x. By accumulating these gradients to obtain the final measure of integrated gradients(IG). More precisely, integrated gradients are defined as the path integral of the gradients along the straight-line path from the baseline x' to the input x.

The integrated gradient along the i-th dimension for an input x and baseline x' is defined as follows. Here, $\partial F(x)/\partial x_i$ represents the gradient of $F(x)$ along the i-th dimension. The Integrated Gradients(IG) that we have computed are shown in Equation 23.

$$IG_i(x) = (x_i - x_i') \times \int_{\alpha=0}^{1} \frac{\partial F(x' + \alpha \times (x - x'))}{\partial x_i} \mathrm{d}\alpha. \tag{23}$$

4. Mathematical Proves

In this sub-section, we will show the mathematical proves of the theorems mentioned in the main text and the previous section.

4.1. Preceding instructions

Before we prove the theorem, let us first introduce the symbols that will be used in the following paragraphs. We will use capital letters to represent the corresponding random variables. For example, X_t represents the random variable of the micro-state $\boldsymbol{x}_t$ at time t, and Y_{t+1} represents the random variable corresponding to the macro-state $\boldsymbol{y}_{t+1}$. For any random variable X, $\tilde{X}$ represents the same random variable X after intervention. $\hat{X}$ means the prediction for X given by neural networks.

Second, to understand how the intervention on Y_t can affect other variables, a causal graph derived from the framework of NIS and NIS+ and depicts the causal relations among variables is very useful, this is shown in Figure 4. In which, X_t and $\hat{X}_{t+1}$ denote the input random variable and predicted output random variable of the NIS+ on a micro scale, while Y_t and $\hat{Y}_{t+1}$ represent the input and output of f_q on a macro scale. After intervening on Y_t, $\tilde{X}_t$, $\tilde{X}_{t+1}, \tilde{Y}_t, \tilde{Y}_{t+1}$ represent different micro or macro variables under the intervention $do(Y_t \sim U(Y))$, corresponding to X_t,$\hat{X}_{t+1}, Y_t$, and $\hat{Y}_{t+1}$, respectively.

4.2. Proof of Theorem 2.1

To prove this theorem, we utilize a combination of the inverse macro-dynamic and probability re-weighting technique, along with information theory and properties of neural networks. In this proof, we need three lemmas, stated as follows:

Lemma 4.1 (Bijection mapping does not affect mutual information)**.** *For any given continuous random variables X and Z, if there is a bijection (one to one) mapping f and another random variable Y such that for any $x \in Dom(X)$ there is a $y = f(x) \in Dom(Y)$, and vice versa, where $Dom(X)$ denotes the domain of the variable X, then the mutual information between X and Z is equal to the information between Y and Z, that is:*

$$I(X; Z) = I(Y; Z), \tag{24}$$

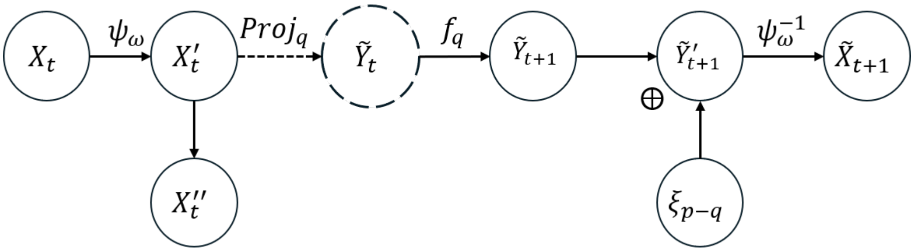

Figure 4. The causal graph among random variables after intervention on Y_t according to the framework of NIS or NIS+. Because the intervention on Y_t can only affect the variables in the upper part of NIS+ framework, we ignore the variables in the lower part. In the diagram, X'_t represents the random variable obtained after reversible transformation of X_t, X''_t represents the variable directly discarded during the projection process, $\tilde{Y}'_{t+1}$ represents a new variable composed of $\tilde{Y}_{t+1}$ concatenated with a standard normal distribution, and ξ_{p-q} represents a $p-q$ dimensional standard normal distribution. The dashed circular shape in the diagram represents the variable that is directly intervened to a uniform distribution, and the dashed arrow represents the causal relationship that is severed due to the intervention.

Lemma 4.2 (Mutual information will not be affected by concatenating independent variables)**.** *If $X \in Dom(X)$ and $Y \in Dom(Y)$ form a Markov chain $X \to Y$, and $Z \in Dom(Z)$ is a random variable which is independent on both X and Y, then:*

$$I(X;Y) = I(X;Y \bigoplus Z). \tag{25}$$

The proofs of Lemma4.2 and Lemma4.1 can be found in the reference [8].

Lemma 4.3 (variational upper bound of a conditional entropy)**.** *Given a conditional entropy $H(\boldsymbol{y}|\boldsymbol{x})$, where $\boldsymbol{x} \in \mathcal{R}^s, \boldsymbol{y} \in \mathcal{R}^q$, there exists a variational upper bound on this conditional entropy:*

$$H(Y|X) \leq -\iint p(\boldsymbol{y},\boldsymbol{x}) \ln g(\boldsymbol{y}|\boldsymbol{x}) \mathrm{d}\boldsymbol{y} \mathrm{d}\boldsymbol{x}, \tag{26}$$

where $g(\boldsymbol{y}|\boldsymbol{x}) \in \mathcal{R}^q \times \mathcal{R}^s$ is any distribution.

Proof. First, we unfold the conditional entropy

$$H(Y|X) = -\iint p(\boldsymbol{x}) p(\boldsymbol{y}|\boldsymbol{x}) \ln p(\boldsymbol{y}|\boldsymbol{x}) \mathrm{d}\boldsymbol{y} \mathrm{d}\boldsymbol{x} \tag{27}$$

Due to the property of KL divergence[11], for any distribution g,

$$D_{KL}(p||g) = \int p(\boldsymbol{x}) \ln \frac{p(\boldsymbol{x})}{g(\boldsymbol{x})} \mathrm{d}\boldsymbol{x} \geq 0. \tag{28}$$

On the other words,

$$\int p(\boldsymbol{x}) \ln p(\boldsymbol{x}) \mathrm{d}\boldsymbol{x} \geq \int p(\boldsymbol{x}) \ln g(\boldsymbol{x}) \mathrm{d}\boldsymbol{x}. \tag{29}$$

So,

$$\begin{aligned} H(\boldsymbol{y}|\boldsymbol{x}) &= -\int p(\boldsymbol{x}) \int p(\boldsymbol{y}|\boldsymbol{x}) \ln p(\boldsymbol{y}|\boldsymbol{x}) \mathrm{d}\boldsymbol{y} \mathrm{d}\boldsymbol{x} \\ &\leq -\int p(\boldsymbol{x}) \int p(\boldsymbol{y}|\boldsymbol{x}) \ln g(\boldsymbol{y}|\boldsymbol{x}) \mathrm{d}\boldsymbol{y} \mathrm{d}\boldsymbol{x} \\ &= -\iint p(\boldsymbol{y},\boldsymbol{x}) \ln g(\boldsymbol{y}|\boldsymbol{x}) \mathrm{d}\boldsymbol{y} \mathrm{d}\boldsymbol{x}. \end{aligned} \tag{30}$$

□

To prove the theorem, we also use an assumption:

Assumption: The composition of the inverse dynamics $g_{\theta'}$ and the encoder ϕ can be regarded as a conditional probability $P(\hat{Y}_t|X_{t+1})$, and this probability can be approximated as a Gaussian distribution $N(g_{\theta'}(\phi(\boldsymbol{x}_{t+1})), \Sigma)$, where $\Sigma = diag(\sigma_1, \sigma_2, \cdots, \sigma_q)$, and σ_i is the MSE loss of the ith dimension of output $\hat{Y}_{t+1}$. Further, suppose σ_i is bounded, thus $\sigma_i \in [\sigma_m, \sigma_M]$ for any i, where σ_m and σ_M are the minimum and maximum values of MSEs.

This assumption is supported by the reference [12].

Next, we restate the theorem that needs to be proved:

Theorem 2.1(Problem Transformation Theorem): For a given value of q, the unconstrained objective function outlined by Eq.(11) serves as the lower bound of constrained objective function defined by Equation 1 of the main text.

Proof. The original constrained goal optimization framework is as follows:

$$\begin{aligned} &\max_{\phi, f_q, \phi^+} \mathcal{J}(f_q), \\ &s.t. \begin{cases} ||\hat{\boldsymbol{x}}_{t+1} - \boldsymbol{x}_{t+1}|| < \epsilon, \\ \hat{\boldsymbol{x}}_{t+1} = \phi^\dagger(f_q(\phi(\boldsymbol{x}_t))). \end{cases} \end{aligned} \tag{31}$$

We know that $\hat{X}_{t+1} = \psi_\omega^{-1}(\hat{Y}_{t+1} \bigoplus \xi)$, where ψ_ω^{-1} is a reversible mapping that doesn't affect the mutual information according to Lemma 4.1. Therefore, based on Lemma 4.2, we have the capability to apply a transformation to the mutual information $I(Y_t, \hat{Y}_{t+1})$:

$$I(Y_t, \hat{Y}_{t+1}) = I(Y_t, \hat{X}_{t+1}). \tag{32}$$

Here, I represents mutual information. By utilizing the property of mutual information, we can derive the following equation:

$$I(Y_t, \hat{X}_{t+1}) = H(Y_t) - H(Y_t|\hat{X}_{t+1}). \tag{33}$$

Now, $H(\tilde{Y}_t) = H(U_q)$, where U_q is a uniform distribution on a macro space. Therefore, we have:

$$\mathcal{J}(f_{\theta,q}) = H(U_q) - H(\tilde{Y}_t|\tilde{X}_{t+1}). \tag{34}$$

The optimization of the objective function $\mathcal{J}(f_q)$ can be reformulated as the optimization of the conditional entropy $H(\tilde{Y}_t|\tilde{X}_{t+1})$, since $H(U_q)$ is a constant. The variational upper bound on $H(\tilde{Y}_t|\tilde{X}_{t+1})$ is obtained by Lemma4.3.

$$H(\tilde{Y}_t|\tilde{X}_{t+1}) \leq -\iint \tilde{p}(\boldsymbol{y}_t, \boldsymbol{x}_{t+1}) \ln g(\boldsymbol{y}_t|\boldsymbol{x}_{t+1}) \mathrm{d}\boldsymbol{y}_t \mathrm{d}\boldsymbol{x}_{t+1}, \tag{35}$$

where $\tilde{p}$ represents the probability distribution function of random variables in case of $\boldsymbol{y}_t$ being intervened.

We will use a neural network to fit the distribution $g(\boldsymbol{y}_t|\boldsymbol{x}_{t+1})$. Based on the assumption, we can consider it as a normal distribution. According to Lemma4.3, since the conditional probability $g(\boldsymbol{y}_t|\boldsymbol{x}_{t+1})$ can be any distribution, we can assume it to be a normal distribution for simplicity. Predicting $\boldsymbol{y}_t$ with $\boldsymbol{x}_{t+1}$ as input can be divided into two steps. First, $\boldsymbol{x}_{t+1}$ is encoded by the encoder ϕ into the macro latent space. Then, the reverse macro dynamics are approximated using $g_{\theta'}$. As a result, the expectation of $g(\boldsymbol{y}_t|\boldsymbol{x}_{t+1})$ can be obtained using the following equation:

$$E_g(\tilde{Y}_t|X_{t+1} = \boldsymbol{x}_{t+1}) \equiv g_{\theta'}(\phi(\boldsymbol{x}_{t+1})) \tag{36}$$

Therefore, we have $g(\boldsymbol{y}_t|\boldsymbol{x}_{t+1}) \sim N(\mu, \Sigma)$, where Σ is a constant diagonal matrix and $\mu = g_{\theta'}(\phi(\boldsymbol{x}_{t+1}))$. In order to utilize the properties of intervention on $\boldsymbol{y}_t$, we need to separate $\tilde{p}(\boldsymbol{y}_t)$. Following Equation 35, we can further transform $H(\tilde{Y}_t|\tilde{X}_{t+1})$:

$$H(\tilde{Y}_t|\tilde{X}_{t+1}) \leq -\iint \tilde{p}(\boldsymbol{y}_t)\tilde{p}(\boldsymbol{x}_{t+1}|\boldsymbol{y}_t)\ln g(\boldsymbol{y}_t|\boldsymbol{x}_{t+1})\mathrm{d}\boldsymbol{y}_t\mathrm{d}\boldsymbol{x}_{t+1}. \tag{37}$$

Assuming that the training is sufficient, we can have

$$\tilde{p}(\boldsymbol{x}_{t+1}|\boldsymbol{y}_t) \approx p(\boldsymbol{x}_{t+1}|\boldsymbol{y}_t). \tag{38}$$

So,

$$H(\tilde{Y}_t|\tilde{X}_{t+1}) \leq -\iint \tilde{p}(\boldsymbol{y}_t)p(\boldsymbol{x}_{t+1}|\boldsymbol{y}_t)\ln g(\boldsymbol{y}_t|\boldsymbol{x}_{t+1})\mathrm{d}\boldsymbol{y}_t\mathrm{d}\boldsymbol{x}_{t+1}. \tag{39}$$

According to Equation36, we obtain the logarithm probability density function of $g(\boldsymbol{y}_t|\boldsymbol{x}_{t+1})$:

$$\begin{aligned}\ln g(\boldsymbol{y}_t|\boldsymbol{x}_{t+1}) &\approx \ln \frac{1}{(2\pi)^{\frac{m}{2}}|\Sigma|^{\frac{1}{2}}} e^{-\frac{(\boldsymbol{y}_t - g_{\theta'}(\phi(\boldsymbol{x}_{t+1})))^2}{2|\Sigma|}} \\ &= -\frac{(\boldsymbol{y}_t - g_{\theta'}(\phi(\boldsymbol{x}_{t+1})))^2}{2|\Sigma|} + \ln \frac{1}{(2\pi)^{\frac{m}{2}}|\Sigma|^{\frac{1}{2}}}.\end{aligned} \tag{40}$$

Because $\ln \frac{1}{(2\pi)^{\frac{m}{2}}|\Sigma|^{\frac{1}{2}}} \geq \ln \frac{1}{(2\pi)^{\frac{m}{2}}|\Sigma|^{\frac{1}{2}}_{max}}$, so

$$\begin{aligned}H(\tilde{Y}_t|\tilde{X}_{t+1}) &\leq \iint \tilde{p}(\boldsymbol{y}_t)p(\boldsymbol{x}_{t+1}|\boldsymbol{y}_t)\left[\frac{(\phi(\boldsymbol{x}_t) - g_{\theta'}(\phi(\boldsymbol{x}_{t+1})))^2}{2|\Sigma|} - \ln \frac{1}{(2\pi)^{\frac{m}{2}}|\Sigma|^{\frac{1}{2}}}\right]\mathrm{d}\boldsymbol{y}_t\mathrm{d}\boldsymbol{x}_{t+1} \\ &\leq \iint \tilde{p}(\boldsymbol{y}_t)p(\boldsymbol{x}_{t+1}|\boldsymbol{y}_t)\left[\frac{(\phi(\boldsymbol{x}_t) - g_{\theta'}(\phi(\boldsymbol{x}_{t+1})))^2}{2|\Sigma|_{min}} - \ln \frac{1}{(2\pi)^{\frac{m}{2}}|\Sigma|^{\frac{1}{2}}_{max}}\right]\mathrm{d}\boldsymbol{y}_t\mathrm{d}\boldsymbol{x}_{t+1},\end{aligned} \tag{41}$$

where $|\Sigma|_{min} = \sigma^q_{min}, |\Sigma|_{max} = \sigma^q_{max}$. Here, $\tilde{p}(\boldsymbol{y}_t)p(\boldsymbol{x}_{t+1}|\boldsymbol{y}_t) = \frac{\tilde{p}(\boldsymbol{y}_t)}{p(\boldsymbol{y}_t)}p(\boldsymbol{x}_{t+1}, \boldsymbol{y}_t)$. Thus, we obtain $\frac{\tilde{p}(\boldsymbol{y}_t)}{p(\boldsymbol{y}_t)}$, where $\tilde{p}(\boldsymbol{y}_t)$ represents the target distribution and $p(\boldsymbol{y}_t)$ represents the natural distribution obtained from the data. We can define the inverse probability weights $w(\boldsymbol{x}_t)$ as follows:

$$w(\boldsymbol{x}_t) \equiv \frac{\tilde{p}(\boldsymbol{y}_t)}{p(\boldsymbol{y}_t)}. \tag{42}$$

Let $z = \frac{(\phi(\boldsymbol{x}_t) - g_{\theta'}(\phi(\boldsymbol{x}_{t+1})))^2}{2|\Sigma|_{min}} - \ln \frac{1}{(2\pi)^{\frac{m}{2}}|\Sigma|^{\frac{1}{2}}_{max}}$. Therefore, we can express Equation 41 as follows:

$$H(\tilde{Y}_t|\tilde{X}_{t+1}) \leq \iint w(\boldsymbol{x}_t)p(\boldsymbol{x}_{t+1}, \boldsymbol{y}_t)z\mathrm{d}\boldsymbol{y}_t\mathrm{d}\boldsymbol{x}_{t+1}. \tag{43}$$

Because we train neural networks using discrete samples $\{x_t\}$, we can use the sample mean as an approximate estimate of the expectation in Equation43. Therefore, the variational upper bound of $H(\tilde{Y}_t|\tilde{X}_{t+1})$ can be written as:

$$H(\tilde{Y}_t|\tilde{X}_{t+1}) \leq \frac{1}{T}\sum_{i=0}^{T-1} w(\boldsymbol{x}_t)z. \tag{44}$$

Substituting the equation into Equation 34, we obtain the variational lower bound of the original objective function:

$$\mathcal{J}(f_{\theta,q}) \geq H(U_q) - \frac{1}{T}\sum_{i=0}^{T-1} w(\boldsymbol{x}_t)z. \tag{45}$$

Therefore, the optimization problem(Equation 1 of the main text) is transformed into

$$\min_{\omega,\theta,\theta'} \sum_{i=0}^{T-1} w(\boldsymbol{x}_t)|\phi(\boldsymbol{x}_t) - g_{\theta'}(\phi(\boldsymbol{x}_{t+1}))|^2 \tag{46}$$

$$s.t.||\hat{\boldsymbol{x}}_{t+1} - \boldsymbol{x}_{t+1}|| < \epsilon, \tag{47}$$

where ω, θ, θ' respectively represent the parameters of the three neural networks ψ, f_θ, $g_{\theta'}$ in the NIS+ framework.

Then we construct Lagrange function,

$$L(\omega, \theta, \theta', \lambda) = \sum_{i=0}^{T-1} w(\boldsymbol{x}_t)|\phi(\boldsymbol{x}_t) - g_{\theta'}(\phi(\boldsymbol{x}_{t+1}))|^2 + \lambda||\phi^\dagger(\boldsymbol{y}_{t+1}) - \boldsymbol{x}_{t+1}|| \tag{48}$$

Therefore, the optimization goal is transformed into

$$\min_{\omega,\theta,\theta'} \sum_{i=0}^{T-1} w(\boldsymbol{x}_t)||\boldsymbol{y}_t - g_{\theta'}(\boldsymbol{y}_{t+1})|| + \lambda||\hat{\boldsymbol{x}}_{t+1} - \boldsymbol{x}_{t+1}|| \tag{49}$$

□

4.3. Proof of Theorem 2.2

We will first use two separate lemmas to prove the scalability of stacked encoder and parallel encoder, thereby demonstrating that their arbitrary combination can keep the conclusion of Theorem 2.1 unchanged.

Lemma 4.4 (Mutual information will not be affected by stacked encoder)**.** *If $X \in Dom(X)$ and $Y \in Dom(Y)$ form a Markov chain $X \to Y$, and Φ_L and $\Phi_L^\dagger$ represent the L-layer stacked encoder and decoder, respectively, then:*

$$I(X;Y) = I(X;\Phi_L^\dagger(Y)). \tag{50}$$

Proof. According to Figure3(a), we can obtain

$$\Phi_L^\dagger(Y) = \phi_{q_1}^\dagger \circ \phi_{q_2}^\dagger \circ \cdots \circ \phi_{q_L}^\dagger(Y). \tag{51}$$

$\phi_{q_i}^\dagger(i = 1, 2, \cdots, L) : \mathcal{R}^{q_i} \to \mathcal{R}^{q_{i-1}}$ is a basic decoder as shown in Equation 10. $\circ$ represents the composition of functions. Therefore, according to Lemma 4.2 and the fact that reversible mappings do not change mutual information, we obtain

$$I(X;Y) = I(X;\phi_L^\dagger(Y)). \tag{52}$$

Let $Y_{L-1} = \phi_L^\dagger(Y)$, we can further obtain $I(X;Y_{L-1}) = I(X;\phi_{L-1}^\dagger(Y_{L-1}))$, and so on, leading to the final result

$$I(X;Y) = I(X;\Phi_L^\dagger(Y)). \tag{53}$$

□

Lemma 4.5 (Mutual information will not be affected by parallel encoder)**.** *If $X \in Dom(X)$ and $Y \in Dom(Y)$ form a Markov chain $X \to Y$, and Φ_T and $\Phi_T^\dagger$ represent parallel encoder and decoder composed of T ordinary encoders or decoders, respectively, then:*

$$I(X;Y) = I(X;\Phi_T^\dagger(Y)). \tag{54}$$

Proof. the decoding process can be divided into two steps:

$$\Phi_T^{\dagger}(Y) = \Psi_T(Y \bigoplus \xi). \tag{55}$$

Y can be decomposed as $Y = Y_1 \bigoplus Y_2 \bigoplus \cdots \bigoplus Y_T$, and all the introduced noise $\xi = \xi_1 \bigoplus \xi_2 \bigoplus \cdots \bigoplus \xi_T$, even if the order of their concatenation changes, it will not affect the mutual information between the overall variable and other variables. Let $\Psi_T(Z) = \psi_1(Z_1) \bigoplus \psi_2(Z_2) \bigoplus \ldots \bigoplus \psi_T(Z_T)$, where $Z_i(i = 1, 2, \ldots, T)$ is a partition of Z, and $\psi_i(i = 1, 2, \ldots, T) : \mathcal{R}^p \to \mathcal{R}^p$ are all invertible functions. Therefore, Ψ_T is also an invertible function, meaning that this function transformation does not change the mutual information. According to Lemma 4.2, we have

$$I(X;Y) = I(X;Y \bigoplus \xi) = I(X;\Psi_T(Y \bigoplus \xi)). \tag{56}$$

Finally proven,

$$I(X;Y) = I(X;\Phi_T^{\dagger}(Y)). \tag{57}$$

□

Next, we provide the proof of Theorem 2.2.

Theorem 2.2(Problem Transformation in Extensions of NIS+ Theorem) When the encoder of NIS+ is replaced with an arbitrary combination of stacked encodes and parallel encoders, the conclusion of Theorem 2.1 still holds true.

Proof. To demonstrate that Theorem 2.1 remains applicable to the extended NIS+ framework, we only need to prove that Equation 32 still holds true, as the only difference between the extended NIS+ and the previous framework lies in the encoder. First, we restate Equation 32 as follows:

$$\mathcal{J}(f_q) = I(Y_t, \hat{Y}_{t+1}) = I(Y_t, \hat{X}_{t+1}). \tag{58}$$

According to Lemma 4.4 and Lemma 4.5, Equation 32 holds true when the encoder of NIS+ is replaced with either a stacked encoder or a parallel encoder. And any combination of these two encoders is nothing more than an alternating nesting of stacking and parallelization, so the conclusion still holds. □

4.4. Proof of Theorem 2.3

To prove the universality theorem, we need to extend the definition of the basic encoder by introducing a new operation $\eta_{p,s} : \mathcal{R}^p \to \mathcal{R}^s$, which represents the self-replication of the original variables.

$$\eta_{p,s}(\boldsymbol{x}) = \boldsymbol{x} \bigoplus \boldsymbol{x}_{s-p}. \tag{59}$$

The vector $\boldsymbol{x}_{s-p}$ is composed of $s-p$ dimensions, where each dimension is a duplicate of a specific dimension in $\boldsymbol{x}$. For example, if $\boldsymbol{x} = (0.1, 0.2, 0.3)$, then $\eta_{2,5}(\boldsymbol{x}) = (0.1, 0.2, 0.3, 0.1, 0.2)$.

The basic idea to prove theorem 2.3 is to use the famous universal approximation theorems of common feed-forward neural networks mentioned in [13, 14] and of invertible neural networks mentioned in [15, 16] as the bridges, and then try to prove that any feed-forward neural network can be simulated by a serious of bijective mapping(ψ), projection(χ) and vector expansion (η) procedures. The basic encoder after the extension for vector expansion can be expressed by the following equation:

$$\phi = Proj_q \circ \psi_s \circ \eta_{p,s} \circ \psi_p. \tag{60}$$

The functions $\psi_s : \mathcal{R}^s \to \mathcal{R}^s$ and $\psi_p : \mathcal{R}^p \to \mathcal{R}^p$ represent two reversible mappings. The final dimensionality q that is retained may be larger than the initial dimensionality p. In the context of coarse-graining microscopic states to obtain macroscopic states, for the sake of convenience, we usually consider ϕ as a dimension reduction operator.

First, we need to prove a lemma.

Lemma 4.6. *For any vector $X \in \mathcal{R}^p$ and matrix $W \in \mathcal{R}^{s\times p}$, where $s,p \in \mathcal{N}$, there exists an integer $s_1 \leq \min(s,p)$ and two basic units of encoder: $\psi_s \circ \eta_{s_1,s}$ and $\chi_{p,s_1} \circ \psi_p$ such that:*

$$W \cdot X \simeq (\psi_s \circ \eta_{s_1,s}) \circ (\chi_{p,s_1} \circ \psi_p)(X), \tag{61}$$

where, $\simeq$ represents approximate or simulate.

Proof. For any $W \in \mathcal{R}^{s\times p}$, we can SVD decomposes it as:

$$W = U \cdot \Lambda \cdot V, \tag{62}$$

where $U \in \mathcal{R}^{s\times s}, V \in \mathcal{R}^{p\times p}$ are all orthogonal matrices, $\Lambda = \begin{pmatrix} diag(\lambda_1, \lambda_2, \cdots, \lambda_{s_1}) & \mathbf{0} \\ \mathbf{0} & \mathbf{0} \end{pmatrix}_{s\times p}$ is a diagonal matrix composed by nonzero eigenvalues of W: $\lambda_i, i \in [1, s_1]$, and zeros, where s_1 is the rank of the matrix W. Because all matrices can be regarded as functions, therefore:

$$W(X) = U(\eta_{s_1,s}(\chi_{p,s_1}(\Lambda' \cdot V)(X))), \tag{63}$$

where, $\Lambda' = diag(\lambda_1, \lambda_2, \cdots, \lambda_{s_1}, 1, \cdots, 1)$. That is, the functional mapping process of $W(X)$ can be decomposed into four steps: matrix multiplication by $\Lambda' \cdot V$, projection χ_{p,s_1}(projecting a p dimensional vector to an s_1 dimensional vector), vector expansion $\eta_{s_1,s}$ (expanding the s_1 dimensional vector to an s dimensional one), and matrix multiplication by U. Notice that the first and the last steps are invertible because the corresponding matrices are invertible. Therefore, according to [15, 16], there are invertible neural networks ψ_s and ψ_p that can simulate orthogonal matrix U and the invertible matrix $\Lambda' \cdot V$, respectively. Thus, $\psi_s \simeq U$, and $\psi_p \simeq \Lambda' \cdot V$. Then we have:

$$(\psi_s \circ \eta_{s_1,s}) \circ (\chi_{p,s_1} \circ \psi_p) \simeq W, \tag{64}$$

That is $W(X)$ can be approximated by an extended stacked encoder. □

With lemma 4.6, we can prove theorem 2.3. We at first restate theorem 2.3 here:

Theorem 2.3(Universal Approximating Theorem of Stacked Encoder): For any continuous function f which is defined on $K \times \mathcal{R}^p$, where $K \in \mathcal{R}^p$ is a compact set, and $p > q \in \mathcal{Z}^+$, there exists an integer s and an extended stacked encoder $\phi_{p,s,q} : \mathcal{R}^p \to \mathcal{R}^q$ with s hidden size and an expansion operator $\eta_{p,s}$ such that:

$$\phi_{p,s,q} \simeq f, \tag{65}$$

Thereafter, an extended stacked encoder is of universal approximation property which means that it can approximate(simulate) any coarse-graining function defined on $\mathcal{R}^p \times \mathcal{R}^q$.

Proof. According to universal approximation theorem in [13, 14], for any function f defined on $K \times \mathcal{R}^p$, where $K \in \mathcal{R}^p$ is a compact set, and $p > q \in \mathcal{Z}^+$, and small number ϵ, there exists an integer s and $W \in \mathcal{R}^{s\times p}, W' \in \mathcal{R}^{q\times s}, b \in \mathcal{R}^s$ such that:

$$W' \cdot \sigma(W + b) \simeq f, \tag{66}$$

where, $\sigma(\boldsymbol{x}) = 1/(1 + \exp(-\boldsymbol{x}))$ is the sigmoid function on vectors.

According to Lemma 4.6 and $+b$ and $\sigma(\cdot)$ are all invertible operators, therefore, there exists invertible neural networks $\psi_q, \psi'_s, \psi_s, \psi_p$, and two integers s_1, s_2 which are ranks of matrices W' and W, respectively, such that:

$$(\psi_q \circ \eta_{s_2,q} \circ \chi_{s,s_2} \circ \psi'_s) \circ (\psi_s \circ \eta_{s_1,s} \circ \chi_{p,s_1} \circ \psi_p) \simeq W' \cdot \sigma(W \cdot +b), \tag{67}$$

where, $\psi_s \circ \eta_{s_1,s} \circ \chi_{p,s_1} \circ \psi_p$ approximates(simulates) the function $\sigma(W \cdot +b)$ and $\psi_q \circ \eta_{s_2,q} \circ \chi_{s,s_2} \circ \psi'_s$ approximates(simulates) the function $W' \cdot$.

Therefore, if we let $\phi_{p,s,q} = (\psi_q \circ \eta_{s_2,q} \circ \chi_{s,s_2} \circ \psi'_s) \circ (\psi_s \circ \eta_{s_1,s} \circ \chi_{p,s_1} \circ \psi_p)$, then $\phi_{p,s,q} \simeq f$ □

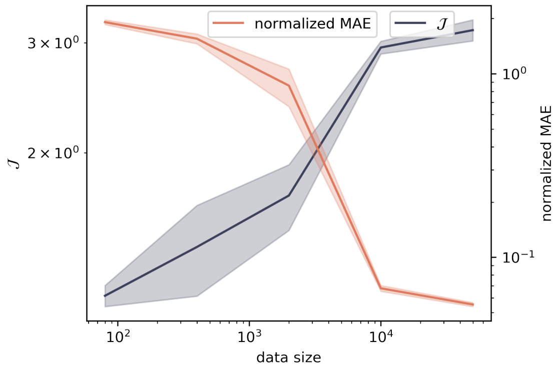

Figure 5. A drop-off in performance with decreasing dataset sizes, meaning that as the volume of data diminishes, $\mathcal{J}$ decreases and the normalized MAE increases.

In real applications, although the basic encoder and the extended versions do not include expansion operator, we always expand input vector before it is input for the encoder. Therefore, it is reasonable to believe that Theorem 2.3 still holds for stacked encoders.

5. Details about the experiments in SIR Model

In this section, we will introduce various parameters and specific methods in the SIR experiment.

5.1. Data generation

In Equation 5 of the main text, $\boldsymbol{\xi}_1, \boldsymbol{\xi}_2 \sim N(0, \Sigma)$ are two-dimensional Gaussian noises and independent of each other, and $\Sigma = \begin{pmatrix} \sigma^2 & -\frac{1}{2}\sigma^2 \\ -\frac{1}{2}\sigma^2 & \sigma^2 \end{pmatrix}$. For this covariance matrix, we can easily deduce that $\boldsymbol{\xi}_1$ and $\boldsymbol{\xi}_2$ are both two-dimensional normal distributions with a correlation coefficient of $-\frac{1}{2}$. The parameter σ controls the overall magnitude of the noise, which is set to $\sigma = 0.03$ in the experiments conducted in this paper. For macroscopic state data, we discretize the original continuous model with $dt = 0.01$ and run 7 time steps for each starting point. After sampling with different starting points, we concatenate equal amounts of $\boldsymbol{\xi}_1$ and $\boldsymbol{\xi}_2$ to obtain four-dimensional microscopic data.

In the SIR model experiment conducted in this article, we generated two datasets: the complete dataset and the partial dataset. For the complete dataset, we uniformly sampled 9000 initial points from the entire range of possible values for S and I ($S \geq 0, I \geq 0, S + I \leq 1$), resulting in a total of 63000 data points. For the partial dataset, we uniformly sampled 6000 initial points from the region where $S \geq \frac{1}{3}$. Additionally, to satisfy the requirements of KDE estimation for the support set, we randomly sampled 10 initial points from the region where $S \leq \frac{1}{3}$. In total, the partial dataset consists of 42070 sample points. The training and testing results of the NIS+ neural network on these two different sample sets are presented in Figure 3 of the main text.

We also assessed the impact of data volume on the outcomes in this experiment. As depicted in Figure 5, the x-axis denotes the size of the dataset, and the two lines represent the trends of $\mathcal{J}$ and the normalized MAE, respectively. The experiment was conducted three times with distinct random seeds. A decrease in the training data leads to a reduction in $\mathcal{J}$ and an increase in the normalized MAE, thereby highlighting the performance of our framework under conditions of sparse data.

5.2. Training and testing details

For NIS+, two stages are separated at the 3000th epoch(see the support information section 2.5). In the second stage (after 3000 epochs), NIS+ incorporates inverse probability weighting and bidirectional dynamic learning, distinguishing it from the NIS model.

In Figure 3(d) of the main text, the test involves selecting 20 starting points within an area where the training samples are missing, evolving them for 10 steps using the learned macro-dynamics, and then decoding the results back into micro-states. The mean of the multi-step prediction errors for these 20 starting points is displayed. In Figure 3(e) of the main text, considering the significant differences in the range of experimental data, the threshold ϵ measures the magnitude of relative error, which is obtained by dividing the absolute error by the standard deviation of the data itself. Figure 6(a) demonstrates a sharp increase in the normalized MAE as σ increases. Within this region, we select a threshold that aligns with the boids experiment. We set $\epsilon = 0.3$, which means that the machine training is considered sufficient when the relative error is less than or equal to 0.3. The selection of the threshold is also unaffected by different values of λ, as can be seen in Figure 6(a). As the λ increases, there is a greater emphasis on optimizing the microstructural reconstruction error, which results in a lower training MAE. However, due to fluctuations, the line is not monotonic, as depicted in Figure 6(b). The objective function $\mathcal{J}$ will decrease as inverse dynamics becomes less critical to maximizing $\mathcal{J}$. We select the value of the parameter λ in a region where there is a balanced comparison between these two factors.

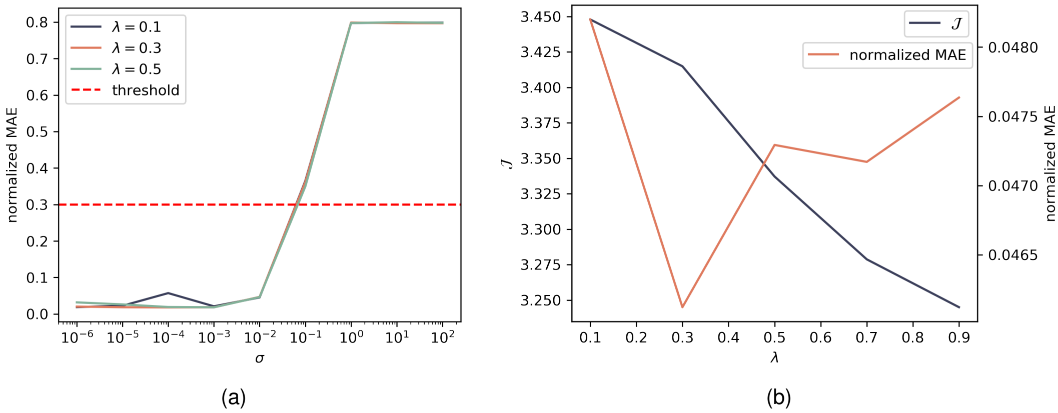

Figure 6. The selection of hyperparameters. (a) illustrates the variation in normalized MAE as the observation noise σ increases across different values of λ. Additionally, the red dashed line represents the threshold $\epsilon = 0.3$ set based on the behavior of the normalized MAE. (b) depicts the changes in $\mathcal{J}$ and the normalized MAE as the λ increases.

5.3. Methods for the vector field results

The following step-by-step explanation outlines how to average the vector field results of emergent dynamics, as demonstrated in Figure 7. The aim of this process is to compute the vector field through extensive sampling and averaging. Initially, we generate grid points on the phase space with a step size of 0.007, as shown in Figure 7(a). This step serves to obtain a significant number of samples for further analysis. To achieve this, we introduce noise to these grid points and their subsequent time steps, extending them to four-dimensional data. Next, we employ the well-trained NIS+ model to encode these extended data points, resulting in samples in the latent space. These samples are then divided into different grids on the latent space with a step size of 0.015, enabling a comprehensive analysis of the emergent dynamics. The subsequent calculation involves determining the average motion vector for each grid using the methodology outlined in Figure 7(d). It is important to note that the primary objective of this step is to compute the average vector field by aggregating the motion vectors across the grids. For comparison, we also generate sparser grid points on the phase space with a step size of 0.07. By applying the same noise and encoding techniques, we establish a one-to-one correspondence between the motion vectors of the grids and the new grid points. This correspondence allows us to depict the vector field of the macro latent space of NIS+ as shown in Figure 3(c) of the main text. Similarly, by replacing the encoder of NIS+ with the encoder of NIS, we can obtain the latent space vector field of NIS, as illustrated in

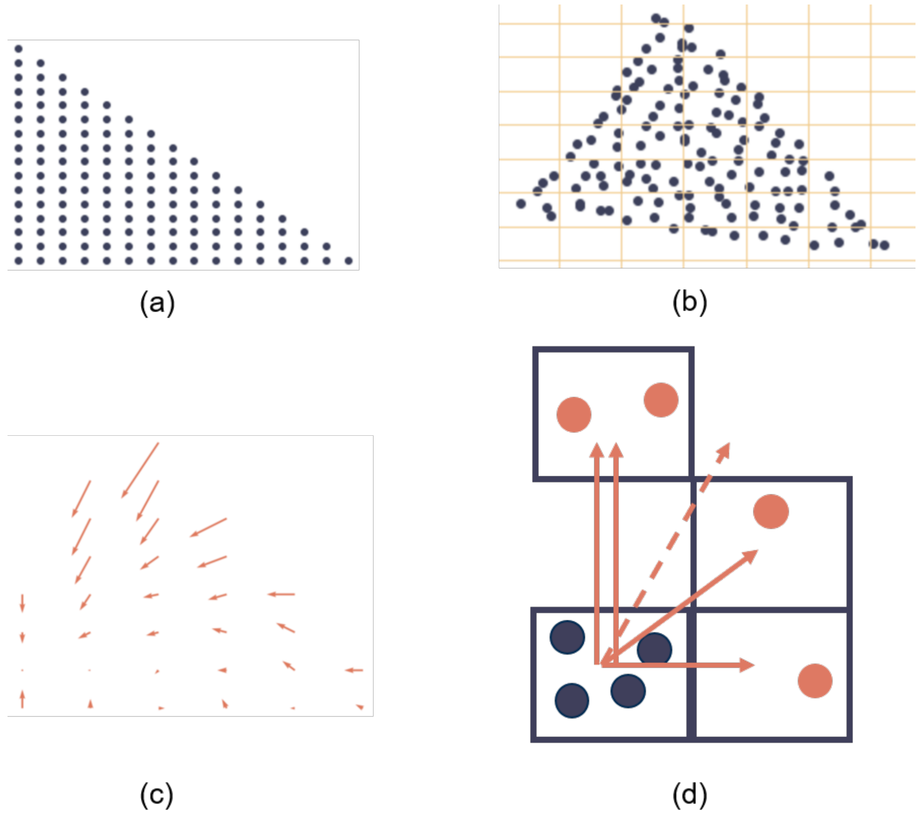

Figure 7. The method of generating the vector fields in Figure 3(c)(f) of the main text. (a) Generate a sufficient number of grid points in phase space. (b) Divide the encoded latent space into grids with certain intervals, and each sample point in the latent space will fall into a certain grid. (c) Take the expectation of different direction vectors for each grid, obtaining the corresponding motion vector for that grid. (d) Zoom in on the local amplification schematic of the averaging process. The black dots in the figure represent the coordinates of sample points at a certain time, and the red dots represent the coordinates of these sample points at the next time step. If a sample point jumps from the i-th grid to the j-th grid in one time step, then a count is made in the direction of i→j, represented by a solid red arrow in the figure. Taking the expectation of all solid arrows yields the motion vector corresponding to that grid, represented by a dashed red arrow.

Figure 3(f) of the main text. This approach, based on extensive sampling and averaging, provides valuable insights into the macro features of emergent dynamics.

In Figure 3(c)(f) of the main text, we provide a theoretic vector field. It is obtained by the folowing steps. First,

$$\frac{d\boldsymbol{y}_t}{dt} = \frac{\partial \phi(\boldsymbol{x}_t)}{\partial t} = \frac{\partial \phi(\boldsymbol{x}_t)}{\partial \boldsymbol{x}_t}\frac{d\boldsymbol{x}_t}{dt}, \tag{68}$$

where $J_\phi \equiv \frac{d\phi(\boldsymbol{x}_t)}{d\boldsymbol{x}_t}$ is the Jacobian matrix of the encoder. Therefore, we could obtain the vector by representing the relationship between the true dynamics and the latent space dynamics. We notice that according to Equation 5 of the main text:

$$\frac{d\boldsymbol{x}_t}{dt} = \frac{d(S_t, S_t, I_t, I_t)}{dt}, \tag{69}$$

Because $\boldsymbol{x}_t = (\boldsymbol{S}'_t, \boldsymbol{I}'_t)$ and the noises are independent of time and have zero mean, we can replace $\frac{d\boldsymbol{x}_t}{dt}$ in the experiment. Finally, we obtain:

$$\frac{d\boldsymbol{y}_t}{dt} = J_\phi \cdot \frac{d(\boldsymbol{S}_t, \boldsymbol{I}_t)}{dt}, \tag{70}$$

where, $\boldsymbol{S}_t = (S_t, S_t)$ and $\boldsymbol{I}_t = (I_t, I_t)$. Therefore, after multiplying the ground truth vector field with the Jacobian matrix J_ϕ, we obtain the theoretic prediction of the vector field in the graph. The difference between this theoretic vector field and the true dynamics is due to the errors of the encoder ϕ.

In order to achieve a better dynamic vector field, we specifically conducted multi-step prediction training when drawing Figure 3(c)(f) of the main text. Specifically, given the input of microstate at a certain moment, we make multi-step predictions for the next 10 microstates respectively and combine them with certain weights to form a loss for gradient backpropagation. The weights decay at a rate of $e^{-0.2t}$ as the prediction steps increase. For reverse prediction, we take the target corresponding to the last step as the input and perform multi-step prediction training in the same way. In addition, we adopt the approach of interval sampling, taking every 100 sample points as a step, so that NIS+ can make long-term predictions on the SIR model.

5.4. Comparison models in the SIR experiment

To compare the advantages of the NIS+ framework itself, we constructed a regular fully connected neural network (NN) with 33,668 parameters and a Variational Autoencoder (VAE) with 34,956 parameters in the SIR experiment (the NIS+ model consists of a total of 37,404 parameters, which includes the inverse dynamics component). NN consists of an input layer, an output layer, and four hidden layers with 4, 64, 128, 128, 64, 4 neurons respectively. NN+ refers to the NN that combines inverse probability weighting technique, with the same parameter framework. For each data point $\boldsymbol{x}_t$, we estimate the KDE probability distribution and calculate the inverse probability weighting. Finally, the weights are multiplied with the loss to give different training weights to samples with different densities.

VAE is a generative model that maps micro-level data to a normal distribution in latent space. During each prediction, it samples from the normal distribution and then decodes it [17]. It consists of an encoder and a decoder, both of which have 4 hidden layers, each layer containing 64 neurons. The input and predicted output are both four-dimensional variables, and the latent space also has four dimensions, with two dimensions for mean and two dimensions for variance. The macro-dynamic learner fits the dynamical changes of the two-dimensional mean variables. During the training process, the loss function consists of two parts: reconstruction error, which is the MAE error between the predicted output and the true label, and the KL divergence between the normal distribution of the latent space samples and the standard normal distribution. These two parts are combined with a 2:1 ratio and used for gradient backpropagation. VAE+ combines inverse probability weighting and bidirectional dynamic learning in the training of VAE, and adopts the same training method as NIS+. We keep the variance variables obtained from encoding unchanged, and use inverse probability weighting to obtain weight factors for the mean variables. Additionally, a reverse dynamic learner is constructed to predict $\boldsymbol{y}_t$ given $\boldsymbol{y}_{t+1}$.

Because NN+ does not have a multi-scale framework for encoding and decoding, it directly estimates and calculates inverse probability weights at the micro-scale data level, and cannot improve the forward dynamics EI through training reverse dynamics. In VAE+, we focus on the dynamics trained in the latent space, aiming to estimate and calculate inverse probability weights in the latent space, and can optimize the encoder by training the reverse dynamics in the latent space to improve EI.The comparison results are shown in Figure 3(b)(d) of the main text.

6. Details in the experiments of Boids Model

In 1986, Craig Reynolds created a simulation of the collective behavior of birds, known as the Boids model [18]. This model only used three simple rules to control the interactions between individuals, resulting in flock-like behavior.

6.1. Method of Simulating the Trajectory of Boids

In Boids model, the micro-state of each individual boid i is a four dimensional vector $(x_i^t, y_i^t, v_{x,i}^t, v_{y,i}^t)$, where $\boldsymbol{x}_i = (x_i, y_i)$ is the positional vector, and $\boldsymbol{v}_i = (v_{x,i}, v_{y,i})$ is the velocity vector. Thus, the

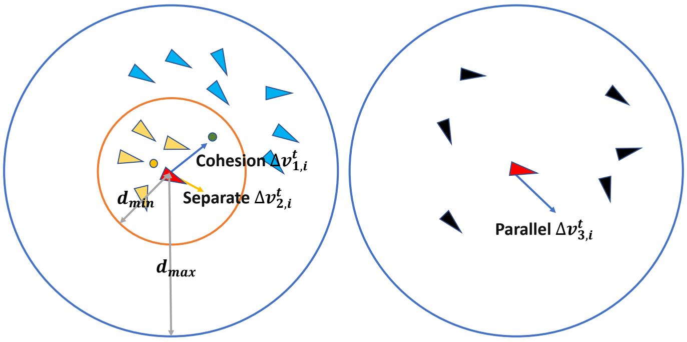

Figure 8. Boids model. By modifying $d_{\max}$ and $d_{\min}$ at time t, the aggregation of bird flocks will change. Specify $\Delta v^t_{1,i}$, $\Delta v^t_{2,i}$, $\Delta v^t_{3,i}$ as the variation of separation, parallelism, and cohesion velocities, respectively. The i-th bird is marked in red. At time t, birds with a distance less than d_{min} from bird i will affect $\Delta v^t_{2,i}$. Birds with a distance less than d_{max} from bird i will affect $\Delta v^t_{1,i}$, $\Delta v^t_{3,i}$. In this way, birds can maintain a distance from each other and allow the Boids to fly in an orderly manner in one direction.

complete micro-state for all N boids is a $4N$ dimensional vector. The micro-states follow the dynamical update equations:

$$\begin{cases} \boldsymbol{x}_i^{t+1} = \boldsymbol{x}_i^t + \boldsymbol{v}_i^t, \\ \boldsymbol{v}_i^{t+1} = \dfrac{\boldsymbol{v}_i^t + \Delta\boldsymbol{v}_i^t + \varepsilon_i^t}{||\boldsymbol{v}_i^t + \Delta\boldsymbol{v}_i^t + \varepsilon_i^t||}||\boldsymbol{v}_i^t||. \end{cases} \tag{71}$$

Where, $\Delta\boldsymbol{v_i}^t$ is the external force exerted on i by its neighbors, and it is composed with three components which called cohesion, alignment, and separation, respectively, according to the dynamical rules of Boids model. $\varepsilon_i^t = (\cos\Delta\alpha_i^t, \sin\Delta\alpha_i^t)^T$ is a force on turning direction exerted on each boid at each time step, where $\Delta\alpha \in [-\pi, \pi]$ is a fixed value or random number.

The motion vector of bird i at time t can be expressed as $(x_i^t, y_i^t, v_{x,i}^t, v_{y,i}^t)$ where x_i^t and y_i^t represent the position coordinates, $v_{x,i}^t$ and $v_{y,i}^t$ represent the projection of velocity on two coordinate axes. With N boids, $i = 1, 2, \ldots, N$. The motion vector can be decomposed into position vector and velocity vector $\mathbf{x}_i^t = (x_i^t, y_i^t)$ and $\mathbf{v}_i^t = (v_{x,i}^t, v_{y,i}^t)$. The distance between two boids i and j can be expressed as $d_{ij} = ||\mathbf{x}_i^t - \mathbf{x}_j^t|| = \sqrt{(x_i - x_j)^2 + (y_i - y_j)^2}$. Although the Boids model belongs to a continuous system, in order to facilitate the simulation process and subsequent experiments, we uniformly set $\Delta t = 1$. For a single boid in the model, the acceleration $\mathbf{a}_i^t = \mathbf{v}_i^{t+1} - \mathbf{v}_i^t$ can be divided into four parts: separation, parallelism, cohesion, and random deflection angle noise. We stipulate that the velocity magnitude of a boid during the flight remains stable, so the impact of acceleration mainly changes the direction of Boids' flight. After knowing the motion state vector of each boid in the flock at time t, the motion state at time $t+1$ can be generated. Specify $\Delta v^t_{1,i}$, $\Delta v^t_{2,i}$, $\Delta v^t_{3,i}$ as the variation of separation, parallelism, and cohesion velocities, respectively. In which

$$\begin{cases} \Delta v^t_{1,i} = \dfrac{\mathbf{x}_i^t - \mathbf{x}_{\Phi_i}^t}{||\mathbf{x}_i^t - \mathbf{x}_{\Phi_i}^t||}, \\ \Delta v^t_{2,i} = \dfrac{\sum_{k\in\Psi_i^t}\mathbf{v}_k^t}{||\sum_{k\in\Psi_i^t}\mathbf{v}_k^t||}, \\ \Delta v^t_{3,i} = -\dfrac{\mathbf{x}_i^t - \mathbf{x}_{\Psi_i}^t}{||\mathbf{x}_i^t - \mathbf{x}_{\Psi_i}^t||}. \end{cases} \tag{72}$$

We stipulate that the maximum and minimum detection distances for Boids are $d_{\max}$ and $d_{\min}$. The sets of Boids within the minimum and maximum detection range are specified as $\Phi_i^t = \{j|d_{ij}^t < d_{\min}\}$ and $\Psi_i^t = \{j|d_{ij}^t < d_{\max}\}$. So the centers of gravity of Φ_i and Ψ_i are represented as

$\mathbf{x}^t_{\Phi_i} = \sum_{k\in\Phi^t_i} \mathbf{x}^t_k/|\Phi_i|$ and $\mathbf{x}^t_{\Psi_i} = \sum_{k\in\Psi^t_i} \mathbf{x}^t_k/|\Psi_i|$, which are the mean values of the boids' position coordinates within two sets. With equation

$$\Delta \boldsymbol{v}^t_i = \Delta v^t_{1,i} + \Delta v^t_{2,i} + \Delta v^t_{3,i} \tag{73}$$

we can obtain Equation 71 in Section 3.2 of the main text. The relationship between the above variables is shown in Fig.8. By modifying the starting vector $(x^0_i, y^0_i, v^0_{x,i}, v^0_{y,i})$ or $d_{\max}$ and $d_{\min}$, the aggregation of bird flocks will change. And we can adjust the trajectory and randomness of Boids by modifying the mean and variance of the deflection angle ε^t_i in Equation 71.

6.2. Methods for Collecting Experimental Data and Training

We record the velocity vector and position vector of each bird at each time t during its flight as data. For each time step t, we need to record the data at that time and the data at the next time step $t+1$. We merge the data into $4N$ dimensional vectors at each time step t as

$$X_t = (x^t_1, y^t_1, v^t_{x,1}, v^t_{y,1}, \ldots, x^t_N, y^t_N, v^t_{x,N}, v^t_{y,N}). \tag{74}$$

This vector corresponds to our micro-state data X_t, $p = 64$. We can repeat the preparation and recording stages multiple times until the data volume meets our expectations. The ID of birds is $1, 2, ..., 16$, evenly divided into two sets, $\{1, 2, \ldots, 8\}$ and $\{9, 10, \ldots, 16\}$. The model we generated contains two sets of birds randomly generated near the center of the canvas.

We generate observation data in two phases: preparation and recording. In the preparation phase, one group of birds is randomly generated within a circle with $(148, 150)$ as the center and 5 as the radius and the other group of birds is randomly generated within a circle with $(152, 150)$ as the center and 5 as the radius. Then by modifying the $(v^0_{x,i}, v^0_{y,i})$ or the mean and variance of the deflection angle ε^t_i two groups of birds can fly different trajectories in Figure 4(a)(4) of the main text. The norm of the velocity vector is always 1, that is $||\mathbf{v}^t_i|| \equiv 1, t = 1, 2, \ldots$. We set $d_{max} = 5$ and $d_{min} = 1$ so that the birds within the same group will interact with each other, while the effects between birds in different groups can be ignored. Subsequently, we allowed two groups of birds to fly for 20 steps without affecting each other, resulting in two groups of birds with distance and relatively stable internal stability. Then the positions and velocities of all birds serve as the starting point for us to record data.

In the recording phase, we have the birds continue to run 50 steps on the basis of the preparation phase, recording the velocity and position vectors of each bird separately. We randomly sort the generated data and input them into our NIS+ model for training, using the multistory structure of the NIS+ in Fig.3 during the training process. We sample the data for 4 batches. After inputting X_t, the microstate Y_t, Y_{t+1} and predicted value $\hat{X}_{t+1}$ can be output, and training can begin. The encoders and decoders are stacked, and the dynamics learners in different layers, $q = 64, 32, 16, 8, 4, 2, 1$, operate in parallel. This design facilitates simultaneous training for different dynamics learners with distinct dimensions and enables parallel searching for the optimal dimension, which is $q = 8$. Due to the previous experiments showing that the model performs better when $q = 8$ than other scales, and having too many layers can also slow down learning efficiency, we can discard layers with $q < 8$ in the later experiments. All the boids are separated into two groups by forcing their $\Delta\alpha^t_i$ as two distinct values $\Delta\alpha^t_i \sim U(0.0058\pi, 0.0098\pi)$ for boids with $i \leq 8$, and $\Delta\alpha^t_i \sim U(-0.0124\pi, -0.0084\pi)$ for boids $i > 8$. Therefore, the two groups will have separating trajectories with different turning angles as shown in Figure 4(a) of the main text.

After training with 800,000 epochs by NIS, $\mathcal{J}_q$ reaches its maximum value when $q = 8$. We continue to optimize the existing model using NIS+ training 400,000 epochs, the results are shown in Figure 4(c) of the main text. Then we can obtain multi-step prediction data as shown in Figure 4(a) of the main text. It can be seen that the multi-step predicted trajectory matches the flight trajectory of the real bird flock. However, if the variance of ε^t_i is large, the training effect of the model will deteriorate, and the multi-step prediction distance will become shorter as shown in Figure 4(e) of the main text. Causal emergence occurs for all tested dimensions(q) (see Figure 4(c) of the main text). With up to $q = 8$ dimensional macro-state vectors, NIS+ can best capture the emergent collective flying behaviors of the two groups by tracing their centers of the trajectories.

This can be visualized by decoding the predicted macro-states into the predicted micro-states as shown in Figure 4(a) of the main text by the two solid lines. With the method of generating data, we can observe the impact of noise. For different observation noises, in cases where the noise is not too significant, we can use the same method to train the model with NIS first, then optimize it with NIS+ to obtain Figure 4(f) of the main text. For different deflection angle noises, after training from random initial parameter values, in order to unify the control variables, we optimized them in the dimension of $q = 8$ and obtained Figure 4(g) of the main text.

In Figure 4(d) of the main text, we highlight the most significant corresponding micro-states for each macro-state dimension with orange dots, determined using the integrated gradients (IG) method applied to our model. The horizontal axis represents the and coordinates of 16 boids in the microscopic state, while the vertical axis represents the 8 macroscopic dimensions. The 1st, 2nd, 5th, and 6th dimensions in macro-states correspond to the boids in the first group (with ID<8), while the 3rd, 4th, 7th, and 8th dimensions correspond to the second group (with ID>=8). We can speculate that there is one bird as a representative in the horizontal and vertical coordinates of the two groups of birds, and observing the position of that bird can predict the overall coordinates. For example, the 10-th bird which is in the second group corresponds to its horizontal axis in the 3-rd and 7-th macro-states, denoted as and , which reflect the overall horizontal axis of group 2. The difference between them can also reflect the direction of motion.

We test the degree of Ψ using the same learned macro-state variable of NIS+. The results, shown in Figure 9, display results of Ψ for different extrinsic noises. For Ψ, all cases yield values are far less than 0. One possible reason to explain this is that much redundant information is ignored by the approximation by Ψ. Another possible reason is that the Boids' coordinate data has a large order of magnitude, which can result in a significant increase in the value of lost information. Thus, unreasonable results are produced which makes Ψ impossible to determine whether CE occurs. Therefore, the proposed $\Delta\mathcal{J}$ in this paper is a superior method for identifying CE.

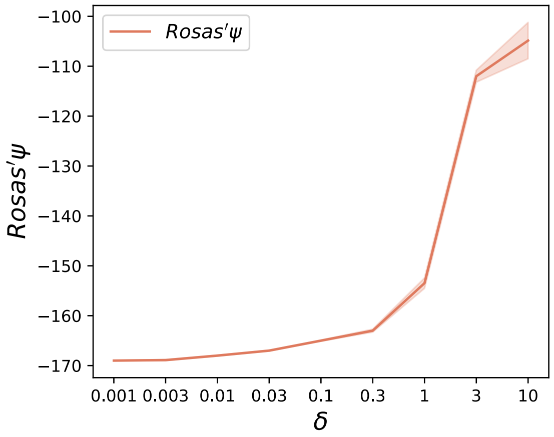

Figure 9. The results of Ψ for different extrinsic noises. For Ψ, all cases yield values are far less than 0. Thus, unreasonable results are produced which makes Ψ impossible to determine whether CE occurs. Therefore, the proposed $\Delta\mathcal{J}$ in this paper is a superior method for identifying CE for Boids model.

7. Game of Life

Conway's Game of Life is a famous two dimensional cellular automota model on which various interesting dynamical patterns like glider, square, flower, signal light, honeycomb, traffic light emerge. Different from SIR model and Boids model, the micro-states of Game of Life at each time step are discrete(0 or 1) on a large regular grid as shown in Figure 10. Further, the micro-dynamics can not be represented by differential or difference equations but by rule tables (details can be referred to the support information section 7.1).

We train NIS+ using data generated by the Game of Life simulation with random initial conditions and extract the time series of states from the 100th step to the 120th step. Figures 10(a),(b) and (c) show the dynamical patterns that are generated by the ground truth simulations(the first row) and the predictions by NIS+(the third row), as well as the emergent macro-states that can make those predictions(the second row). We input two images with successive time steps into NIS+, and obtain another image pair with next two successive time steps. Compare the upper picture and lower ones, the patterns are similar. However, the learned and predicted patterns in the third column, specifically the “glider” pattern, appear vague due to the limited occurrence of training samples with this pattern in random initial conditions. To enhance the quality of predictions, we can generate a new set of training samples that include initial conditions with two “gliders”. As a result, the predictions become clearer, as depicted in Figure 10(d), even though the number of gliders in this test environment is three. That means, NIS+ can capture the patterns including moving, static, and oscillating structures.

Furthermore, we evaluate the generalization ability of the models in test environments that differ from the training environments. We compare the multi-step prediction performance of NIS and NIS+ across eight different pattern types, which are distinct from the initial random patterns. The results are depicted in Figure 10(f) showing that NIS+ consistently achieves a higher AUC (area under the curve) than NIS for all the pattern types. Where, in the tick labels of the x-coordinate, we adopt the format of “pattern name (quantity)” to represent various initial conditions. For instance, “glider(2)” signifies an initial configuration comprising two gliders. Thus, we have provided the evidence that the enhanced NIS+ method possesses superior generalization ability in capturing these patterns.

We further test the degree of CE ($\Delta\mathcal{J}$) and compare it with Ψ using the same learned macro-state variable of NIS+. We use the same patterns as initial conditions to test for the comparison. The results, shown in Figure 10(g), display results of CE, in which each bar representing a combination of $\Delta\mathcal{J}$ and Ψ for one initial pattern. Regarding $\Delta\mathcal{J}$, except for the “random” case, all eight cases demonstrate the occurrence of CE. The case with “glider” patterns exhibit the lowest degree of CE due to poor prediction (see Figure 10(c)). The remaining seven patterns show similar $\Delta\mathcal{J}$ values. These results indicate that $\Delta\mathcal{J}$ provides a more reasonable indication of the occurrence of CE, aligning with our intuition. However, for Ψ, all cases yield values less than or equal to 0. One possible reason to explain this is that much redundant information is ignored by the approximation by Ψ. Thus, unreasonable results are produced which makes Ψ impossible to determine whether CE occurs. Therefore, the proposed $\Delta\mathcal{J}$ in this paper is a superior method for identifying CE.

Additionally, this example highlights the versatility of NIS+. In order to conduct the aforementioned experiments, we needed to coarse-grain the micro-states of the cellular automata in both spatial and temporal dimensions. To address this challenge, we incorporated the concept of spatiotemporal convolution. The architecture used in this experiment is illustrated in Figure 10(e). The entire coarse-graining process can be divided into two steps: first, aggregating information within a fixed-size window (a 3x3 window in this paper) to obtain spatial coarse-grained results; and second, aggregating these results over multiple successive time steps to form a spatiotemporal coarse macro-state. All of these processes are implemented through parallel encoders in NIS+.

7.1. Data generation

In this paper, we use Conway’s Game of Life as the experimental object, in which each cell has two states for two-dimensional state input: alive (1) or dead (0), and each cell is affected by its eight neighbors. The evolution of the Game of Life is only affected by the input state and its update rules, in which the Game of Life has four evolutionary rules, respectively corresponding to cell reproduction and death, and so on. The update rules for the Game of Life are shown in the following table:

The training sample generation process of the Game of Life is as follows: firstly, a state x_t is initialized. When considering a temporal coarse-grained of two steps, the subsequent three steps of states x_{t+1}, x_{t+2}, and x_{t+3} are then generated based on the update rule and are input to the machine learning model. The two input states are x_t and x_{t+1}, with the micro-dynamics output

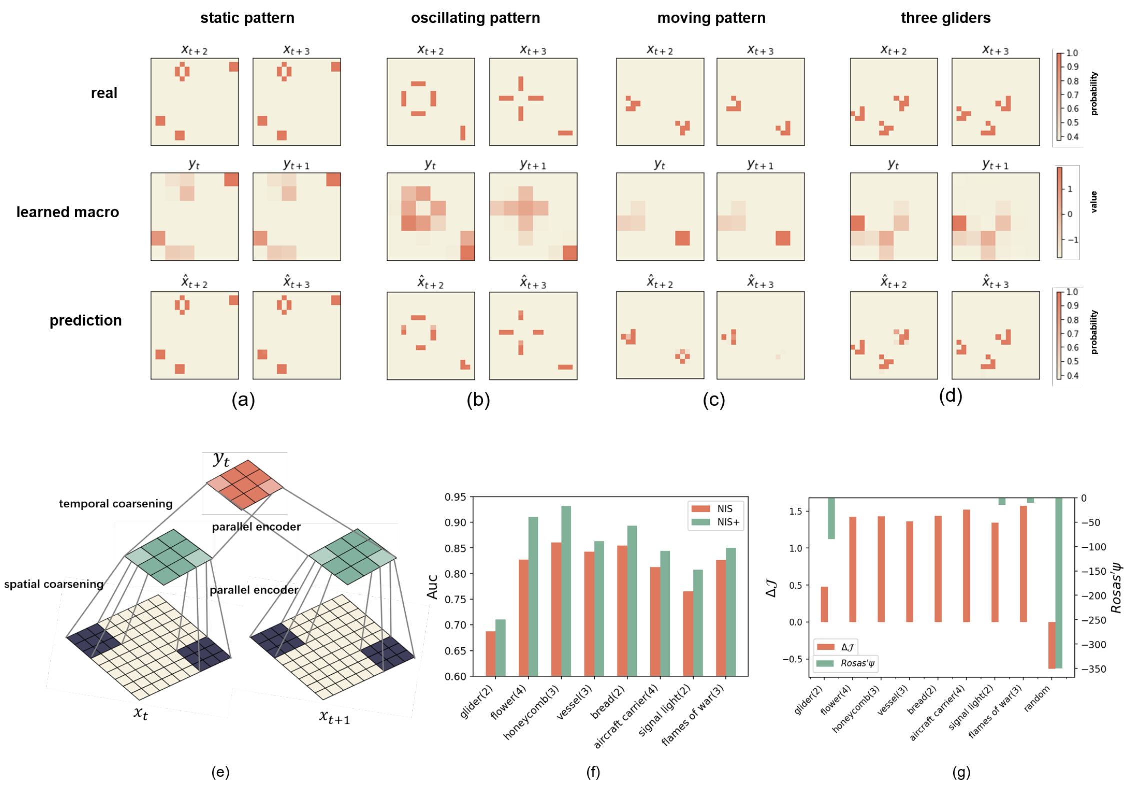

Figure 10. Experiments on the Game of Life. (a-d) depict the comparisons between the ground truth simulations, learned macro-states, and the predictions made by NIS+ for different patterns. In (a-c), the training data is generated by simulating the Game of Life for 120 steps with random initial conditions, and only the images from steps 100-120 are selected to form the training set. (d) depicts a generalization test involving the number of gliders was conducted, transitioning from the original input of two gliders to three gliders. In (a-d), the predicted images are the averaged results obtained by sampling from the predicted macro-states using the decoder 20 times. The colors represent the probability of repetitions in the 20 samples. (e) The architecture of the encoder is illustrated, which can be divided into spatial encoding (mapping 3x3 grids to 1 grid) and temporal encoding (mapping 2 time steps to 1 step) stages. These processes are implemented using parallel encoders. (f) shows the comparisons of AUC (area under the curve) between NIS and NIS+ on different patterns, where each result is the average of multi-step predictions (2 steps). Each case represents multiple quantity combinations of a selected pattern as the initial states. There are 9 patterns, which can be divided into four categories: static (flower, vessel, bread, aircraft carrier and honeycomb), oscillating (signal light, flames of war), moving (glider), and random (random), The horizontal coordinate corresponding to each bar in the figure is represented by the format of "pattern name (quantity)". (g) Comparisons of the degrees of CE ($\Delta\mathcal{J}$, dimension averaged EI and Ψ) are tested with various patterns.

Table 1. The update rules for the Game of Life.

x_t	x_{t+1}
0	1 (there are three living cells around)
1	0 (less than two surviving cells around the cell (excluding two))
	1 (there are two or three living cells around)
	0 (there are more than three living cells around)

being x_{t+1} and x_{t+2}. Due to the utilization of spatiotemporal coarse-graining, the macro-dynamics will output a macro-state which will be decoded into the micro-states x_{t+2} and x_{t+3}. This process is repeated multiple times (50,000 samples) and generate the data for training in Figure 10d. While in the other experiments, we generate 500,000 samples.

7.2. The model predictive ability on glider pattern

We then test the capability of capturing dynamical patterns on glider pattern, where the model was trained based on two glider patterns. The model depicts a good prediction effect and the results are shown in Figure 11.

In addition, please refer to Table 2 for more detailed information on the other model parameters.

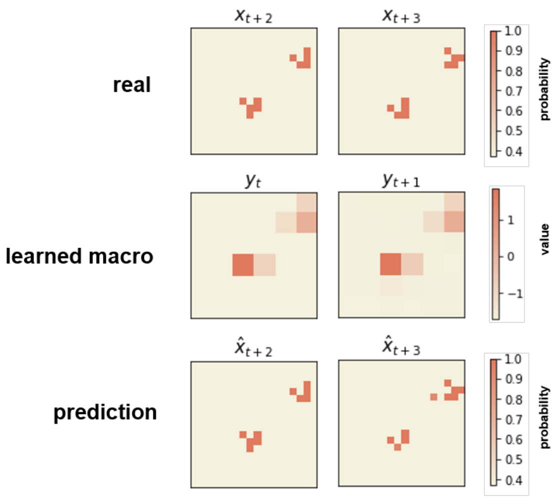

Figure 11. **Experiments of Game of Life.** The results show the dynamical patterns that are generated by the ground truth simulations(the first row) and the predictions by NIS+(the third row), as well as the emergent macro-states that can make those predictions(the second row). Here the input state size of the model test is 18×18×2 and also consists of two gliders.

8. Details about the experiments in fMRI

8.1. About resting state

We also apply integrated gradient to see what seven macro dimensions mean for micro brain areas. Different from results from visual fMRI,we can see some distinct patterns. Unfortunately, we can't see that some one-to-one relationship between these seven dimensions and seven systems defined by Schaefer atlas(See Figure 12)

8.2. AAL Results

We repeat what we have done in Section 3.3 of the main text and support information section 8.3 expect that we have utilized AAL3 atlas and made λ to be 0.5 for layer 1 training of NIS+ since $\lambda = 1$ will lead to a trivial solution where macro predictions for all areas will remain the same, which make the prediction can't be meaningful. We can see a converging evidence that NIS+ framework will lead to an improvement of $\Delta\mathcal{J}$(see Figure 13(b) and a stronger correlation with those areas which responds to the visual areas(See Figure 13(a) and (c)).

8.3. Pre-processing fMRI time series data in detail

When working with fMRI data, the high dimensionality of the data (over 140,000 dimensions) poses computational challenges. To address this, dimension reduction techniques through brain

atlas are employed to make the calculations feasible. We first preprocessed the data by removing the effects of artificial motion, global signal, and white matter signal and cerebrospinal fluid signal using fmriPrep results contained in AOMIC ID1000 [19, 20]. Simultaneously, we detrend and normalize the data during this process. By doing the whole preprocessing process, we also throw out 51 subjects' data since some time steps of these subject's have been removed. Furthermore, to facilitate the investigation of the correlation between the required brain function for watching movie clips and the emergent macro dynamics, we employed the Schaefer atlas to divide the brain into 100 functional areas. Also, we have committed the philosophy of anatomical Atlas, which seems to suggest the converging evidence(see support information section 8.2). All of these preprocessing steps were performed using Nilearn[21, 22]. During the training process, the only difference is the use of PCA in dimension reduction to handle the KDE approximation required for reweighting if $q > 10$.

8.4. Compare with other framework for identifying causal emergence

The figure presented in Figure 14 illustrates our experimentation with the NIS+ framework for identifying causal emergence using both fMRI data from movie-watching sessions and resting-state fMRI data, as detailed in Section 1.2 [5]. For the q=1 model, the Ψ values are relatively high for both visual fMRI and resting fMRI data, while none exceed 0. Here, "micro data" refers to the average original visual fMRI dataset across subjects, while "macro data" encompasses insights derived from the NIS+ framework across subjects. Different from NIS+, the macro data typically reflects behavioral patterns in the past experiment of[5]. For instance, the micro data comprises electrocorticography (ECOG) data recorded during macaques' wrist movements, while the macro data offers an approximation of this movement behavior. In contrast, the macro data derived from NIS+ is obtained by achieving a balance between minimizing predictive errors and maximizing effective information, without relying on behavioral data. This approach potentially provides advantages over other frameworks for identifying causal emergence since the behavior data is not required for testing this framework and it seems to show more sensitivity to properties behind dataset as it can detect the difference between visual fMRI and resting fMRI dataset.

9. The Structure and parameters of neural networks.

Next, we list the neural network parameters used in our experimental study, as shown in Table 2. The parameter λ in the table is derived from Equation 11 and serves as the weight coefficient for the forward prediction error. L is an assumption about the range of data distribution, which represents the length of a hypercube with side length $2L$ that is used to calculate the probability value for a uniform distribution. It is not difficult to see that different types of neural networks have been used to handle different types of complex systems. Both the Boids and fMRI experiments employ the Multistory NIS+ framework, which traverses different macroscopic scales and eventually selects an optimal scale (the dimension of input or output in the Inverse Dynamics Learner table).

Table 2. Parameter table for all experiments conducted using neural networks.

Experiment	Module	Parameter
SIR	Encoder(Decoder)	Three RealNVP modules<br>Input(Onput) dimensions: 4<br>Macro-state dimensions: 2
	Dynamics Learner	Input(Onput) dimensions: 2<br>Hidden Layers: 2<br>Hidden Units: 64<br>Activation Function: LeakyReLu<br>Total Epochs:30000<br>Batch Size:700<br>L: 1
	Inverse Dynamics Learner	λ: 0.33<br>Input(Onput) dimensions: 2<br>Hidden Layers: 2<br>Hidden Units: 64<br>Activation Function: LeakyReLu<br>Stage II Epochs:27000
Boids	Encoder(Decoder)	Three RealNVP modules<br>Input(Onput) dimensions: 64<br>Macro-state dimensions: 32,16,8
	Dynamics Learner	Input(Onput) dimensions: 64,32,16,8<br>Hidden Layers: 2<br>Hidden Units: 32<br>Activation Function: LeakyReLu<br>Total Epochs:800,000<br>Batch Size:4<br>L: 100
	Inverse Dynamics Learner	λ: 1<br>Input(Onput) dimensions: 8<br>Hidden Layers: 2<br>Hidden Units: 32<br>Activation Function: LeakyReLu<br>Stage II Epochs:400,000
Game of life	Encoder(Decoder)	Three RealNVP modules<br>Input(Onput) dimensions: $18 \times 18 \times 2$<br>Macro-state dimensions: $6 \times 6 \times 1$
	Dynamics Learner	Input(Onput) dimensions: 36<br>Hidden Layers: 2<br>Hidden Units: 64<br>Activation Function: LeakyReLu<br>Total Epochs: 300,000<br>Batch Size: 50<br>L: 100
	Inverse Dynamics Learner	λ: 1<br>Input(Onput) dimensions: $18 \times 18 \times 2$<br>Hidden Layers: 2<br>Hidden Units: 64<br>Activation Function: LeakyReLu<br>Stage II Epochs: 200,000
fMRI	Encoder(Decoder)	Six RealNVP modules<br>Input(Onput) dimensions: 100<br>Macro-state dimensions: 52,27,14,7,3,1
	Dynamics Learner	Input(Onput) dimensions: 100,52,27,14,7,3,1<br>Hidden Layers: 5<br>Hidden Units: 256<br>Activation Function: LeakyReLu and a final tanh<br>Total Epochs: 60000<br>Batch Size: 100<br>L: 1
	Inverse Dynamics Learner	λ: 1<br>Input(Onput) dimensions: 1<br>Hidden Layers: 5<br>Hidden Units: 256<br>Activation Function: LeakyReLu Stage II Epochs: 10000

References

[1] E. P. Hoel, L. Albantakis, and G. Tononi, "Quantifying causal emergence shows that macro can beat micro," *Proceedings of the National Academy of Sciences*, vol. 110, no. 49, pp. 19 790–19 795, 2013.

[2] L. R. Goldberg, "The book of why: The new science of cause and effect," *Notices of the American Mathematical Society*, p. 1, Aug 2019. [Online]. Available: http://dx.doi.org/10.1090/noti1912

[3] P. L. Williams and R. D. Beer, "Nonnegative decomposition of multivariate information," *arXiv preprint arXiv:1004.2515*, 2010.

[4] P. A. Mediano, F. Rosas, R. L. Carhart-Harris, A. K. Seth, and A. B. Barrett, "Beyond integrated information: A taxonomy of information dynamics phenomena," *arXiv preprint arXiv:1909.02297*, 2019.

[5] F. E. Rosas, P. A. M. Mediano, H. J. Jensen, A. K. Seth, A. B. Barrett, R. L. Carhart-Harris, and D. Bor, "Reconciling emergences: An information-theoretic approach to identify causal emergence in multivariate data," *PLOS Computational Biology*, vol. 16, no. 12, p. e1008289, Dec 2020. [Online]. Available: http://dx.doi.org/10.1371/journal.pcbi.1008289

[6] C. Lu and J. Peltonen, "Enhancing nearest neighbor based entropy estimator for high dimensional distributions via bootstrapping local ellipsoid," in *Proceedings of the AAAI Conference on Artificial Intelligence*, vol. 34, no. 04, 2020, pp. 5013–5020.

[7] L. Dinh, J. Sohl-Dickstein, and S. Bengio, "Density estimation using real nvp," *International Conference on Learning Representations,International Conference on Learning Representations*, May 2016.

[8] J. Zhang and K. Liu, "Neural information squeezer for causal emergence," *Entropy*, vol. 25, no. 1, p. 26, 2022.

[9] M. Rosenblatt, "Remarks on some nonparametric estimates of a density function," *The annals of mathematical statistics*, pp. 832–837, 1956.

[10] M. Sundararajan, A. Taly, and Q. Yan, "Axiomatic attribution for deep networks," *Cornell University - arXiv,Cornell University - arXiv*, Mar 2017.

[11] S. Fang and Q. Zhu, "Independent gaussian distributions minimize the kullback-leibler (kl) divergence from independent gaussian distributions." *Cornell University - arXiv*, Nov 2020.

[12] C. Blundell, J. Cornebise, K. Kavukcuoglu, and D. Wierstra, "Weight uncertainty in neural network," in *International conference on machine learning.* PMLR, 2015, pp. 1613–1622.

[13] K. Hornik, "Approximation capabilities of multilayer feedforward networks," *Neural Networks*, vol. 4, no. 2, pp. 251–257, 1991.

[14] S. Haykin, *Neural Networks: A Comprehensive Foundation, Volume 2.* Prentice Hall, 1998.

[15] T. Teshima, I. Ishikawa, K. Tojo, K. Oono, M. Ikeda, and M. Sugiyama, "Coupling-based invertible neural networks are universal diffeomorphism approximators," in *Advances in Neural Information Processing Systems*, vol. 33, 2020, p. 3362–3373.

[16] T. Teshima, K. Tojo, M. Ikeda, I. Ishikawa, and K. Oono, "Universal approximation property of neural ordinary differential equations," *arXiv preprint arXiv:2012.02414*, 2017.

[17] D. P. Kingma and M. Welling, "Auto-encoding variational bayes," *arXiv preprint arXiv:1312.6114*, 2013.

[18] C. W. Reynolds, "Flocks, herds and schools: A distributed behavioral model," in *Proceedings of the 14th annual conference on Computer graphics and interactive techniques*, 1987, pp. 25–34.

[19] O. Esteban, C. Markiewicz, R. Blair, C. Moodie, A. Isik, A. Erramuzpe, J. Kent, M. Goncalves, E. DuPre, M. Snyder, H. Oya, S. Ghosh, J. Wright, J. Durnez, R. Poldrack, and K. Gorgolewski, "fmriprep: a robust preprocessing pipeline for functional mri," *Nature Methods*, vol. 16, 01 2019.

[20] O. Esteban, R. Ciric, K. Finc, R. Blair, C. Markiewicz, C. Moodie, J. Kent, M. Goncalves, E. DuPre, D. Gomez, Z. Ye, T. Salo, R. Valabregue, I. Amlien, F. Liem, N. Jacoby, H. Stojić, M. Cieslak, S. Urchs, and K. Gorgolewski, "Analysis of task-based functional mri data preprocessed with fmriprep," *Nature Protocols*, vol. 15, 06 2020.

[21] F. Pedregosa, G. Varoquaux, A. Gramfort, V. Michel, B. Thirion, O. Grisel, M. Blondel, P. Prettenhofer, R. Weiss, V. Dubourg, J. Vanderplas, A. Passos, D. Cournapeau, M. Brucher, M. Perrot, and E. Duchesnay, "Scikit-learn: Machine learning in Python," *Journal of Machine Learning Research*, vol. 12, pp. 2825–2830, 2011.

[22] A. Abraham, F. Pedregosa, M. Eickenberg, P. Gervais, A. Mueller, J. Kossaifi, A. Gramfort, B. Thirion, and G. Varoquaux, "Machine learning for neuroimaging with scikit-learn," *Frontiers in neuroinformatics*, vol. 8, p. 14, 02 2014.

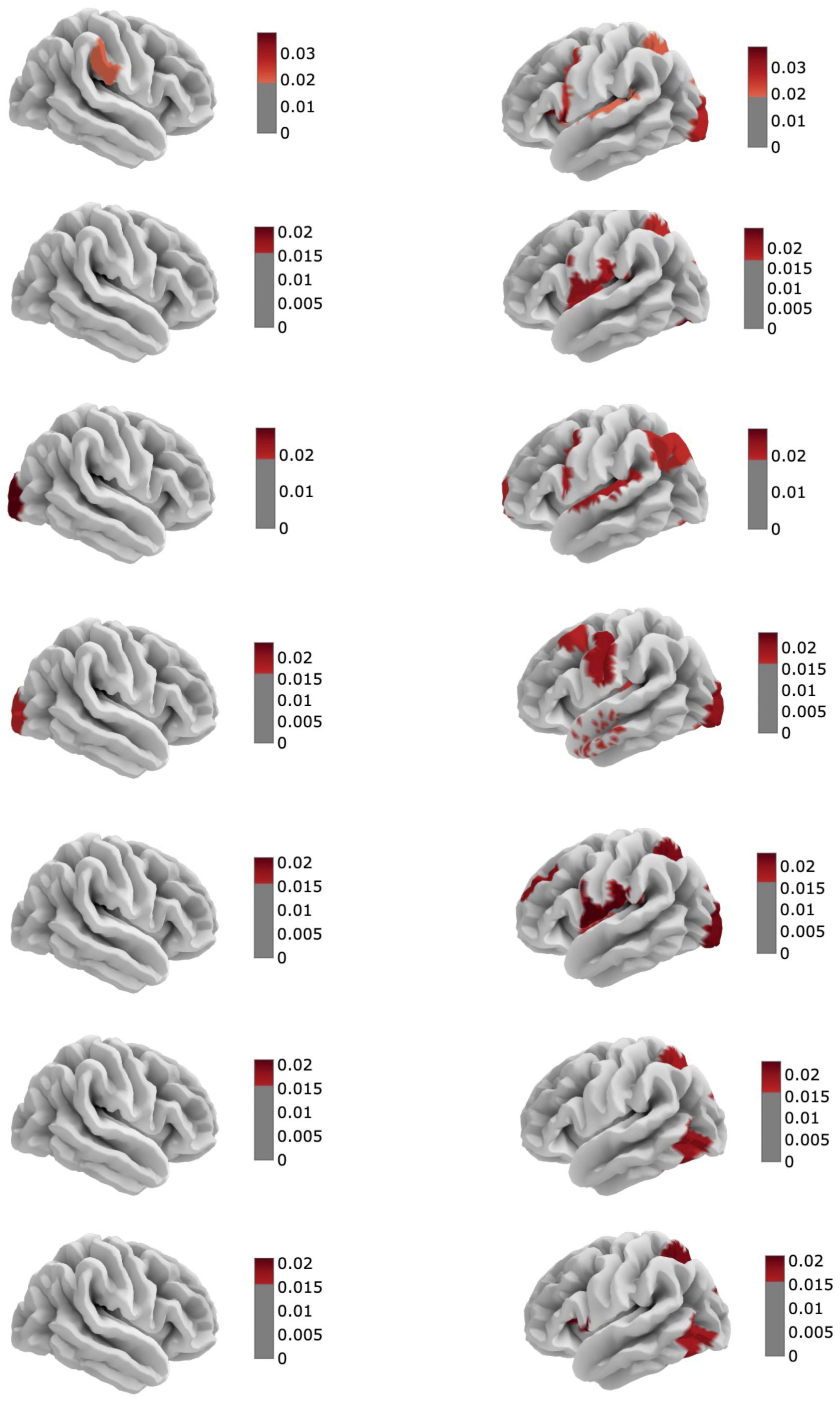

Figure 12. The integrated gradient results in resting NIS+ analysis when $q = 7$.Each pair of left hemisphere and right hemisphere correspond to one macro dimension.Only five percent of most important areas in attribution value are drawn to better show the distinct patterns between seven different marco dimenions

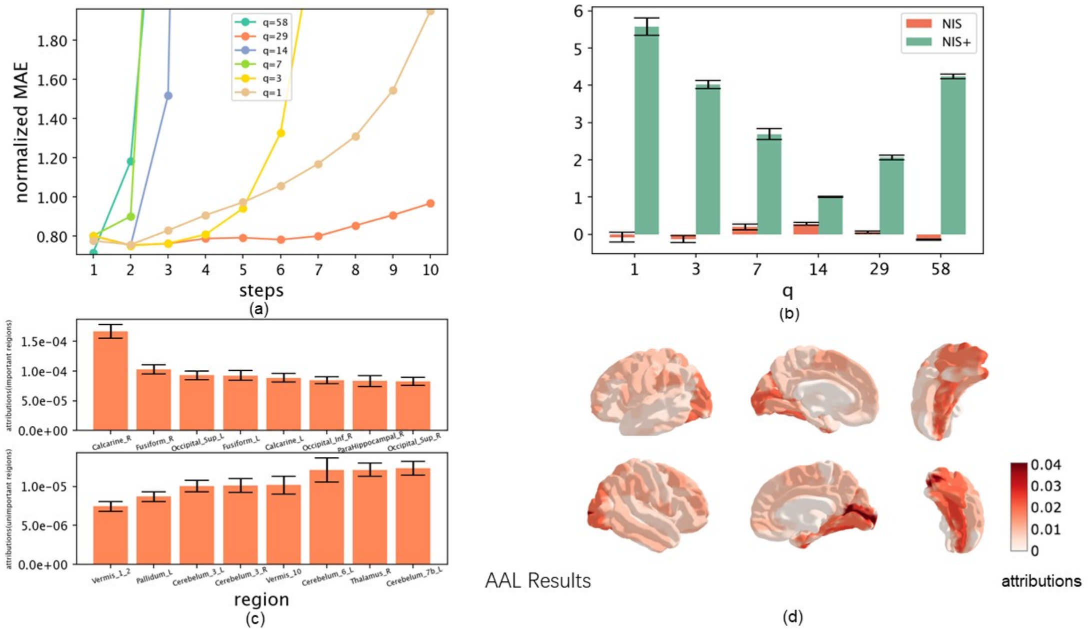

Figure 13. The learning results, the degree of causal emergence, and the attribution analysis of NIS+ and NIS on the fMRI data for the brains. (a) The mean errors of the multi-step predictions increase with the prediction steps under different scales (q) on the test dataset. (b) Measures of CE (dimension averaged, $\Delta\mathcal{J}$) are compared among different models and different datasets including movie-watching fMRI (visual fMRI) and resting fMRI. The bars show the averaged results for 10 repeating experiments, and the error bar represents the standard deviation. (c) The attributions of top 8 significant and insignificant areas under the AAL Atlas are presented. The error bar represents the standard errors. (d) Attribution maps for movie-watching(visual) fMRI data are displayed. The maps show the left hemisphere from the view of the left, left hemisphere from the view of the right, right hemisphere from the view of the right, and right hemisphere from the view of the left. Also, the right column reflects a detailed map for visual areas. The upper is left visual areas and the bottom is right visual areas. The colors represent the normalized absolute values of the integrated gradient.

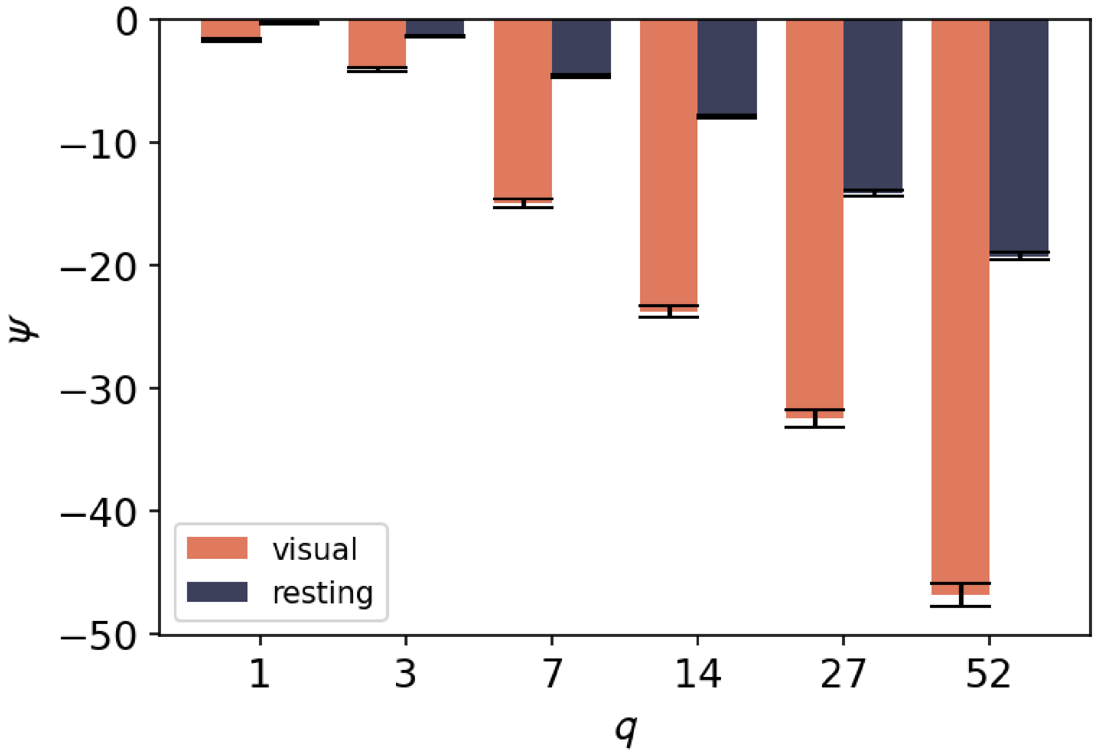

Figure 14. The bar plot to depict Ψ versus scale q for visual fMRI and resting fMRI. As q get lowers, Ψ will increase. However, none of them exceeds 0.
